# Exodex Adam—A Reconfigurable Dexterous Haptic User Interface for the Whole Hand

**DOI:** 10.3389/frobt.2021.716598

**Published:** 2022-03-03

**Authors:** Neal Y. Lii, Aaron Pereira, Julian Dietl, Georg Stillfried, Annika Schmidt, Hadi Beik-Mohammadi, Thomas Baker, Annika Maier, Benedikt Pleintinger, Zhaopeng Chen, Amal Elawad, Lauren Mentzer, Austin Pineault, Philipp Reisich, Alin Albu-Schäffer

**Affiliations:** ^1^ Institute of Robotics and Mechatronics, German Aerospace Center (DLR), Wessling, Germany; ^2^ Faculty of Mechanical Engineering, Munich University of Applied Science, Munich, Germany; ^3^ Faculty of Informatics, Technical University of Munich, Munich, Germany; ^4^ Department of Informatics, Faculty of Mathematics, Informatics and Natural Science, University of Hamburg, Hamburg, Germany; ^5^ Department of Electrical Engineering, Chalmers University of Technology, Göteborg, Sweden; ^6^ Department of Computer Science and Electrical Engineering, Stanford University, Stanford, CA, United States

**Keywords:** haptic user interface, hand exoskeletons, human–machine interface (HMI), human–robot interface (HRI), teleoperation

## Abstract

Applications for dexterous robot teleoperation and immersive virtual reality are growing. Haptic user input devices need to allow the user to intuitively command and seamlessly “feel” the environment they work in, whether virtual or a remote site through an avatar. We introduce the DLR Exodex Adam, a reconfigurable, dexterous, whole-hand haptic input device. The device comprises multiple modular, three degrees of freedom (3-DOF) robotic fingers, whose placement on the device can be adjusted to optimize manipulability for different user hand sizes. Additionally, the device is mounted on a 7-DOF robot arm to increase the user’s workspace. Exodex Adam uses a front-facing interface, with robotic fingers coupled to two of the user’s fingertips, the thumb, and two points on the palm. Including the palm, as opposed to only the fingertips as is common in existing devices, enables accurate tracking of the whole hand without additional sensors such as a data glove or motion capture. By providing “whole-hand” interaction with omnidirectional force-feedback at the attachment points, we enable the user to experience the environment with the complete hand instead of only the fingertips, thus realizing deeper immersion. Interaction using Exodex Adam can range from palpation of objects and surfaces to manipulation using both power and precision grasps, all while receiving haptic feedback. This article details the concept and design of the Exodex Adam, as well as use cases where it is deployed with different command modalities. These include mixed-media interaction in a virtual environment, gesture-based telemanipulation, and robotic hand–arm teleoperation using adaptive model-mediated teleoperation. Finally, we share the insights gained during our development process and use case deployments.

## 1 Introduction

With our hands, we can communicate (read Braille, make gestures, or speak sign language), explore the world around us (feel surface impedances, textures, weights, temperature, pressure), manipulate it, and mold it. For all these functionalities, the somatosensory system of the human is essential. This includes the knowledge about the orientation and position of our body in space (proprioception) and the sense of motion in our joints (kinesthesia) as well as perception of sensory signals from the mechanoreceptors in our skin (cutaneous perception) (Hannaford and Okamura, 2016). All these senses contribute to our ability to receive haptic feedback when interacting with the environment.

In recent years, we have become increasingly used to interacting with remote or virtual environments visually and auditorily, considering voice or video calling, and virtual reality headsets or video games. Haptic interaction is still less widespread but is gaining more interest as haptic technology develops.

This article presents our novel dexterous haptic hand–arm user interface (UI) concept and the development of the Exodex Adam (referred to simply as Exodex for the remainder of the article). Using our preliminary concept for a UI as the starting point (Lii et al., 2017), we developed and integrated various features to make the Exodex a safe and functional haptic user input system for the whole hand. It takes the form of a front-facing, mirror attachment system connected to the user’s fingers and palm. Thanks to its dexterous robotic fingers, each with three actuated degrees of freedom (DOF), joint torque, and angular position sensing, the Exodex is able to render omnidirectional force reflection to the fingertips and the palm triggering mechanoreceptors in the skin. The palm interaction not only allows the pose and joint configuration of the hand to be accurately determined but also allows force reflection during power grasps and whole-hand exploration. A dexterous robotic arm can be mounted on the Exodex to extend the user’s workspace, as well as additional force reflection and gravity compensation. Furthermore, as human hands come in a variety of sizes and shapes, the Exodex can be adjusted through eight reconfigurable mechanisms for the desired fit.

The user is attached to the Exodex at each attachment point through a passive 3-DOF gimbal with low-friction ball bearings. Magnetic clutches ensure safe detachment in case of excessively high torques during operation. Safety is a critical part of the physical human–robot interaction (pHRI). While no pHRI can be perfectly safe, the Exodex is designed to minimize the chance of injury. The mirroring design means that the mechanical parts do not go between the human fingers, reducing the risk of pinching or clamping.

In the next section, we visit the state of the art in dexterous haptic UIs, showing how our device addresses previously unexplored challenges. We then detail the design concepts and developed system device in Section 3 and describe the process for obtaining the best workspace through the placement of attachment positions and configuration adjustments in Section 4. We discuss the optimization of the device kinematics to best suit the set of positions expected of the human hand in Section 5. We detail the low-level control of the Exodex in Section 6: how friction and inertia are reduced, and how the human’s position is estimated for accurate haptic rendering. Section 7 evaluates our system’s effectiveness in different teleoperation modalities through deployment in several use cases: in whole-hand perception of a virtual environment, gesture-commanded telemanipulation, and adaptive model-mediated teleoperation (MMT) of a hand–arm avatar robot. Finally, Section 8 concludes with our closing thoughts on Exodex’s design and deployment, as well as looking to the work ahead.

## 2 Related Work

Our hands are our most capable instruments for intuitively exploring and manipulating the environment. To enable such intuitive interactions in the virtual or in remote environments, hand exoskeletons and haptic UI have been developed, which can track the finger kinematics and reflect reaction forces of the environment back to the user.

### 2.1 Haptic User Interface and Exoskeleton Designs for the Hand

The first commercially available hand exoskeleton, the CyberGrasp, was introduced in the 1990s. It is a tendon-driven device that applies (uni-directional) tensile forces to the human fingers and has been used in many applications and different iterations (Lii et al., 2010; Aiple and Schiele, 2013). The CyberGrasp System continues to serve as a benchmark for the development of new systems. The HaptX Glove goes a step further by adding tactile feedback to force feedback at the fingertip, to introduce cutaneous perception. This is realized by the addition of a custom-designed textile laid out with micro-fluidic channels that can be actuated to press against the user’s skin at commanded locations (Goupil et al., 2019). Some use cases do not require the whole hand or all the fingers for interaction. The PERCRO dual-finger exoskeleton has 3-DOF mechanisms each for the thumb and index finger. It can provide up to 5 N of force at the fingertip. A 3-DOF force sensor is implemented to measure user feedback at each finger (Fontana et al., 2009). The Rutgers Master II (Bouzit et al., 2002) is another such hand exoskeleton attached to the thumb and three fingers of the user and is driven by pneumatic actuators to eliminate the need for tendons and pulleys, such as in the CyberGrasp system. Hall effect and infrared sensors built into the exoskeleton helps track the motion of the operator’s hand. Pneumatic actuators are also implemented in the Festo ExoHand (Festo, 2012).

For coupling between the user’s hand and the haptic UI at the joint level, the Maestro Hand Exoskeleton (Yun et al., 2017) uses a novel mechanism to attach individual actuators and position sensing for each phalanx of the user’s finger. This has been realized into a multi-finger solution. To tackle cost constraints of such systems, the Dexmo (Gu et al., 2016) and HEXOTRAC (Sarakoglou et al., 2016) exoskeletons both aim to be inexpensive and lightweight to make exoskeletons available to a broader market. Dexmo renders haptic feedback through a shifting servo-unit. As a result, the forces can only be displayed in a binary manner. This makes it incapable of rendering more complex object properties, such as stiffness discrimination. The HEXOTRAC employs a different approach to reduce cost, by making it highly under-actuated. It is attached to three of the user’s fingers (the thumb, index, and middle), each with a 6-DOF mechanism driven by a single motor (Gu et al., 2016). The system implements a novel set of kinematics, leaving a wide, natural workspace for each digit. The system is suitable for a wide variety of hand sizes without adjustments being necessary, but this makes it bulky. The force rendering resolution is limited (Sarakoglou et al., 2016).

Another way of realizing in-hand haptic UI is through a mirror attachment solution (e.g., attaching in front of the hand) (Barbagli et al., 2003; Kawasaki et al., 2003; Kawasaki and Mouri, 2007). A full five-fingered Haptic Interface Robot (HIRO) realizing this concept has been presented by Endo et al. (2009) and Endo et al. (2011). HIRO is mounted on a robotic arm making it a grounded device, in contrast to all the aforementioned systems which can be categorized as ungrounded. This is further discussed in the following section.

### 2.2 Grounded Haptic User Interface Devices

Most hand or hand–arm exoskeletons fall in the category of ungrounded devices, which are directly attached to the human hand and body. This makes them flexible and enlarges the workspace of the human user, but comes at the cost of adding the weight of the system to be carried by the human which can lead to fatigue over time. Additionally, with these types of devices, it is impossible to render forces acting on the whole hand, such as reaction forces from large solid/deformable surfaces or immersion in a fluid.

By contrast, grounded devices such as the HIRO (Endo et al., 2011) and the Exodex presented in this article, as well as some non–whole-hand devices such as Force Dimension’s sigma.7 (Tobergte et al., 2011), and the Phantom Omni (Silva et al., 2009), can counter these problems. Since a grounded UI is mounted on a base fixed to the environment, the user need not actively support the weight of the system. This also allows alternative ways of coupling between the exoskeleton and the user’s hand, such as the HIRO’s mirror attachment concept (Endo et al., 2011). It places the UI in front of the user, rather than directly on their appendages.

### 2.3 Haptic Feedback in Teleoperation and Virtual Reality

With haptic feedback, the operator can receive calculated feedback from an environment in virtual reality (VR), whereas in teleoperation, feedback is measured from the remote environment (Stone, 2000). In augmented reality scenarios, where the environment can have an overlay of virtual cues to better assist the user, even a combination of the two is possible (Hedayati et al., 2018).

In all these cases, adding haptic feedback can help improve the user performance in comparison to tasks that were carried out with visual feedback only (Son et al., 2011; Wildenbeest et al., 2012; Weber and Eichberger, 2015). Particularly in the medical field, the augmentation of reality and the implementation of haptic feedback has proven to greatly improve the performance of surgeons, e.g., for minimally invasive surgery situations (Gerovichev et al., 2002).

Since humans are well trained to use their hands for daily interactions, allowing the user to use and receive feedback directly via haptic UI to their hands would be, we expect, more intuitive. The user can explore the virtual or remote environment with natural exploration procedures and intuitive motions, a feature that is increasingly exploited for interactive hand rehabilitation (Missiroli et al., 2019). However, interactions with whole-hand input devices require an estimation of the human hand position and configuration to apply appropriate feedback to multiple areas on the hand. In Endo et al. (2011), the fingertips were tracked and users could manipulate a simple virtual object in precision grasp with force feedback to the fingertips. Although coupled to the user only at the fingertips, rather than the whole hand, the HaptX glove with tangible tactile sensors was shown to teleoperate a Shadow robotic hand (Goupil et al., 2019).

### 2.4 Moving Toward Shared Control for Haptic Hand and Hand–Arm User Interface

Pairing shared control strategies with a haptic UI enables user interaction at different levels of immersion or abstraction, which allows the operator to choose the most effective mode of teleoperation for given tasks. To date, a haptically coupled hand exoskeleton has rarely been applied in such a fashion.

However, the desire and success for shared control capability has been seen with other UI devices. This was especially observed in several space telerobotics experiments. In Kontur-2 and METERON SUPVIS Justin, both carried out from the International Space Station (ISS) to ground, DLR’s dexterous humanoid robot Justin (Fuchs et al., 2009) was commanded using a 2-DOF force-reflection joystick (Artigas et al., 2016) and task-driven supervised autonomy based GUI (Schmaus et al., 2019), respectively, to perform a variety of dexterous robotic tasks. Although the ISS crew members in both experiments were able to successfully complete their given tasks, they have expressed the desire for different UI modalities to be available for more effective teleoperation (Lii et al., 2018).

In light of this, the Analog-1 experiment demonstrated the first successful UI console on board the ISS, combining a GUI, open-loop joystick, and the aforementioned Force Dimension sigma.7 haptic input device. The UI console was used to command a dual-arm rover on ground to perform driving and sample return tasks (Krueger et al., 2020). A similar approach of shared control was also applied for home elderly care using an intuitive GUI for task level command and a dual-arm haptic input device for more dexterous unplanned tasks (Vogel et al., 2020).

As previously mentioned, hand-based shared control teleoperation, particularly with haptic feedback, has been rare. Lii et al. (2012) succeeded in combining joint-level, Cartesian-level, and gesture/task-level teleoperation into a robotic hand grasping and manipulation strategy, albeit without haptic feedback. It nonetheless reduced user mental and physical workload, while improving task success rate.

## 3 Design Concept and Realization

**FIGURE 1:**
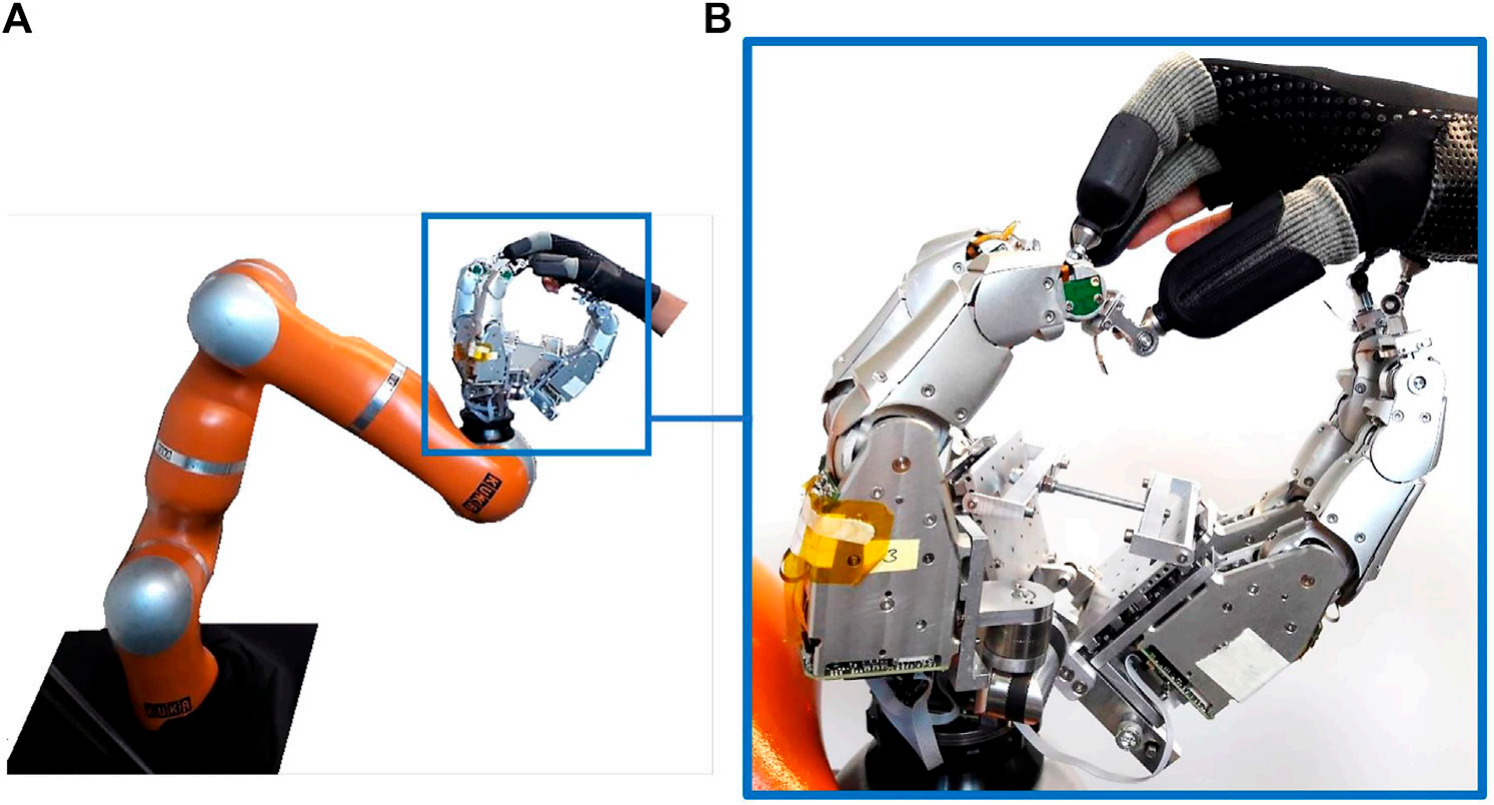
The Exodex whole-hand haptic interface. With multiple robot fingers attached to the fingertips and palm of the user, it is capable of capturing the user’s hand pose in real-time **(B)**. A DLR LWR arm **(A)** greatly increases the available workspace for the user to teleoperate robotic avatars or in virtual reality.

The Exodex’s main design aim is to create an immersive haptic user input device for the whole hand. We introduce a front-facing, mirror-attachment haptic robotic hand UI with a total of 22 active DOF, with an additional eight passive user-reconfigurable DOF to accommodate most hand sizes and shapes. This section describes our overall design concept, which enables Exodex to capture the hand pose and provide whole-hand user immersion with force reflection. Furthermore, a number of features have been developed to help achieve our design goal. These features are also detailed here, including the dexterous robotic finger, reconfigurable palm, robotic arm implementation, user attachment system, as well as safety design. A view of the overall system can be seen in Figure 1. An overview of the system specification is given in Table 1.

**TABLE 1:** Overview of Exodex specifications.

Modular robotic fingers	
Total fingers	5
Total actuated DOF	15 (3 per finger)
Range of motion	±20° abduct/adduct (base joint)
	5°–85° flexion (base joint)
	5°–85° flexion (1:1 coupled distal/medial joint)
Max. joint velocity	180°/sec
Max. force	10 N (at TCP)
Sensing	Joint torque sensor and angular position sensor at each joint
Robotic arm	
Total actuated DOF	7
Range of motion	±170° (joints 1, 3, 5, 7)
	±120° (joints 2, 4, 6)
Max. joint velocity	112.5°/sec (except joint 5)
	180°/sec (joint 5)
Max. force	130 N (at TCP)
Sensing	Joint torque sensor and angular position sensor at each joint
Passive reconfigurable DOF (in the palm base)	
Sliding adjustment	
Total DOF for manual adjustment	4 (at the base of each attached robotic finger)
Rotational adjustment	
Total DOF for manual adjustment	3 (co-located at the base of robotic finger for the user’s thumb)
Palm base cupping angle adjustment	
Total DOF for manual adjustment	1

DOF, degrees of freedom; TCP, tool center point.

### 3.1 Overall Concept to Realize Immersive Whole-Hand Interaction

Unlike most hand exoskeleton and haptic UIs implementations that are fitted over the back of the hand (Lii et al., 2010; Endo and Kawasaki, 2014), the proposed design of Exodex employs a front-facing, mirror attachment design, with the system in front of the user’s hand. Exodex aims to achieve several features that differ from existing haptic hand UI designs. To deliver a safe, immersive haptic experience, the Exodex (see Figure 1) combines the following features into a novel package:

• whole-hand haptic experience for the fingers and the palm surface,
• hand pose estimation capability,
• reconfigurability to accommodate most or all hand geometry and sizes,
• less interference with user movement,
• easy attachment and detachment, and
• user safety.

The Exodex differs from other front-facing fingertip UIs such as the HIRO (Endo et al., 2009; Endo et al., 2011) in its aim to serve the whole hand of the user, i.e., the fingers and the palm surface. The latter two heavily involve the human palm (Gonzalez et al., 2014; Chabrier et al., 2015). Functionality for haptic feedback to the palm is lacking in the grounded hand exoskeletons cited so far. The Exodex introduces attachment points to the user’s palm with dexterous robotic fingers, as well as the fingertips, as shown in Figure 1. The user’s hand is attached to the Exodex through a magnetic clutch and 3-DOF free-rotating gimbal mechanism. The addition of palm interaction is not merely adding more robotic fingers to the system. Rather, it changes the nature of the UI from a fingertip interface, as introduced in Barbagli et al. (2003), Kawasaki et al. (2003), Kawasaki and Mouri (2007), Endo et al. (2009), and Endo et al. (2011), to a UI for the whole-hand haptic interaction. The holistic haptic interaction enables not only precision grasps and manipulation but also power grasps and whole-hand exploration. As presented by in-hand taxonomies (Cutkosky, 1989; Bullock et al., 2013; Feix et al., 2016), we see that grasping and manipulation of objects involve not only the fingers but also the palm, in many cases. With Exodex’s approach, we have the possibility to reproduce the full in-hand haptic experience.

**FIGURE 2:**
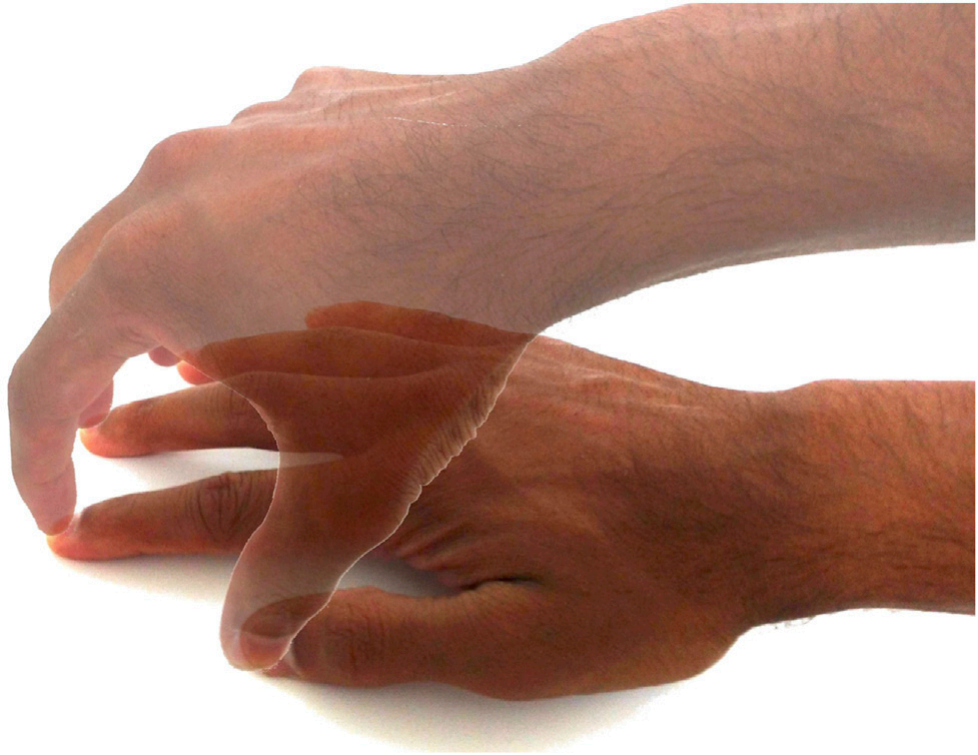
As the two superimposed hands with co-located fingertip positions show, the position of the fingertips alone is not sufficient to determine the pose of the hand.

Furthermore, the Exodex can capture the user’s hand pose without additional sensors being placed directly on the user’s hand. This would not be possible if the user is only attached at the fingertips to a UI device. Figure 2 illustrates this point, where vastly different hand poses having the same fingertip positions. By connecting the user’s palm into the teleoperation, the palm can also become an active contributor to use command input.

Human hands vary widely in geometry and sizes, even when only considering adult users (Greiner, 1991). To serve all of these different hands, an 8-DOF reconfigurable palm base has been developed in the Exodex, which can be configured to fit the vast majority of, if not all, users, including children and adults.

With a device capable of delivering large forces at high speeds, safety against injury is paramount. The front-facing arrangement enables the possibility to implement release mechanisms for safe, fast, detachment to protect the user at any sign of danger. Since the user would be placed in front of, and away from, most of the haptic UI’s mechanisms, they can simply physically pull back to be safely released from the haptic UI and away from its workspace. Finally, to give the user more usable workspace, the Exodex is integrated with a 7-DOF KUKA-DLR Light Weight Robot (LWR) arm (Bischoff et al., 2010) to provide an extension of haptically coupled workspace.

In its current configuration, the Exodex can be used to reconstruct the pose of the human hand except for the ring and little fingers, as they are not yet attached to the system. However, they can be served by adding more robotic fingers to the modular palm base in future implementations. The reconstruction of the use’s hand pose is described in Section 6.

### 3.2 Modular Dexterous Robotic Fingers

The Exodex employs a modular design with self-contained robotic fingers to interact with the user’s hand through angular and force/torque sensing, and force reflection.

**FIGURE 3:**
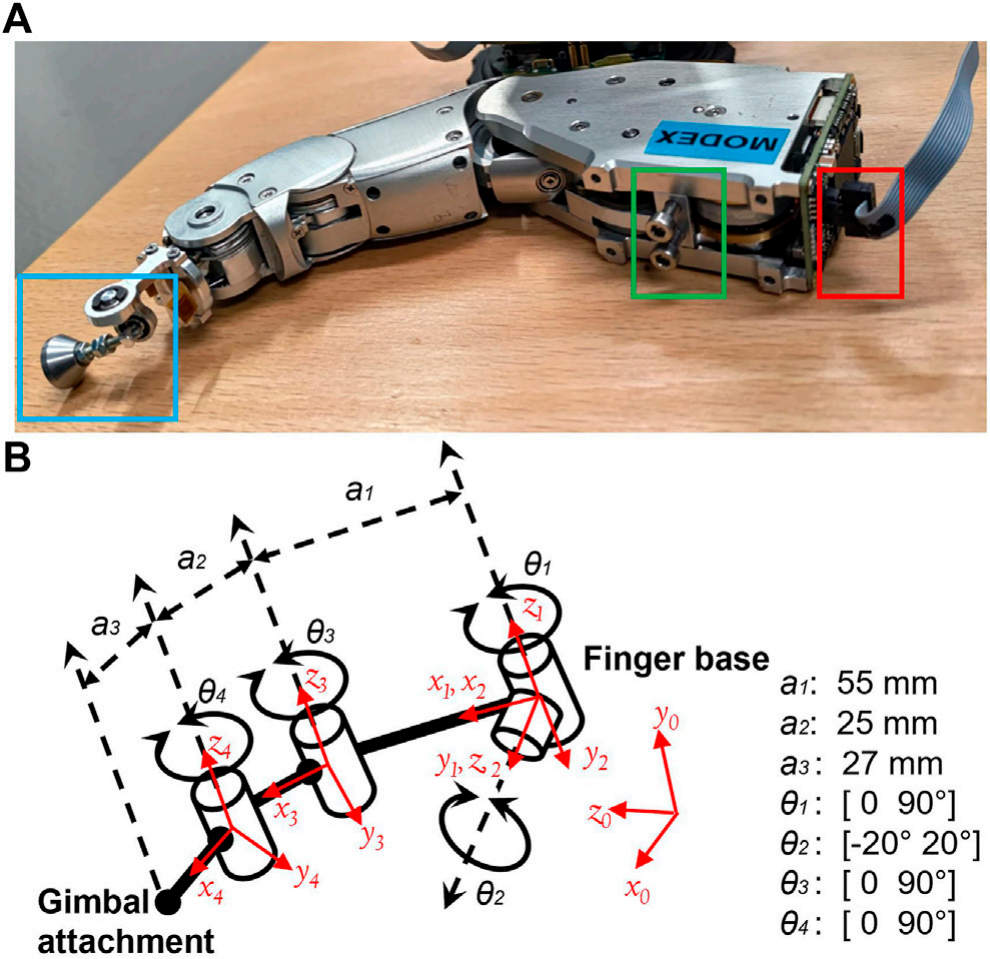
**(A)** The modular robotic finger used in the Exodex. Two screws (marked by the green box), and a single data/power (marked by the red box) cable complete the physical and electrical installation. Each finger is equipped with brushless direct current motors, angular position, and joint torque sensors at each of the three active DOFs. A 3-DOF gimbal (marked in the blue box) enables free rotation at the attachment point to the user. **(B)** The kinematic definition of the robotic finger as modified from a standard DLR Five-Finger Hand. Specifically, the link length has been modified to fit the added gimbal mechanism.

The current version of the Exodex employs customized robotic fingers (see Figure 3) based on those from the DLR Five-Finger Hand (Liu et al., 2008; Chen et al., 2014). Each finger has three DOF, including two active DOF at the base for flexion and abduction/adduction, and an additional 1:1 coupled distal–medial flexion joint. The three active joints of each finger are driven by brushless direct current (BLDC) motors. The joint angular position is measured via a hall effect sensor and potentiometer, whereas the interaction force is measured through a joint torque sensor. Motor command and sensor data are transmitted in a 200 µs cycle through a point-to-point high-speed serial communication at 25 Mbps in a time-triggered fashion.

Its self-contained actuation, sensing, and local data processing enable each finger to serve as a self-contained module. The Exodex fingers can be easily detached and reattached with two screws and a single ribbon data connector, making them easy to replace in the field. For more specifications (see Table 1).

### 3.3 Reconfigurable Modular Palm Base

The human hand come in a large variety of sizes and shapes: from 212 mm for a 95th percentile adult male to 163 mm for a fifth percentile adult female (Greiner, 1991). Therefore, it is advantageous to be able to adjust the distance between the robot fingers attached to the human fingertips and those attached to the palm. This would allow most, if not all, users’ thumb, fingers, and palm surfaces to be in the manipulable workspace of the robotic finger.

**FIGURE 4:**
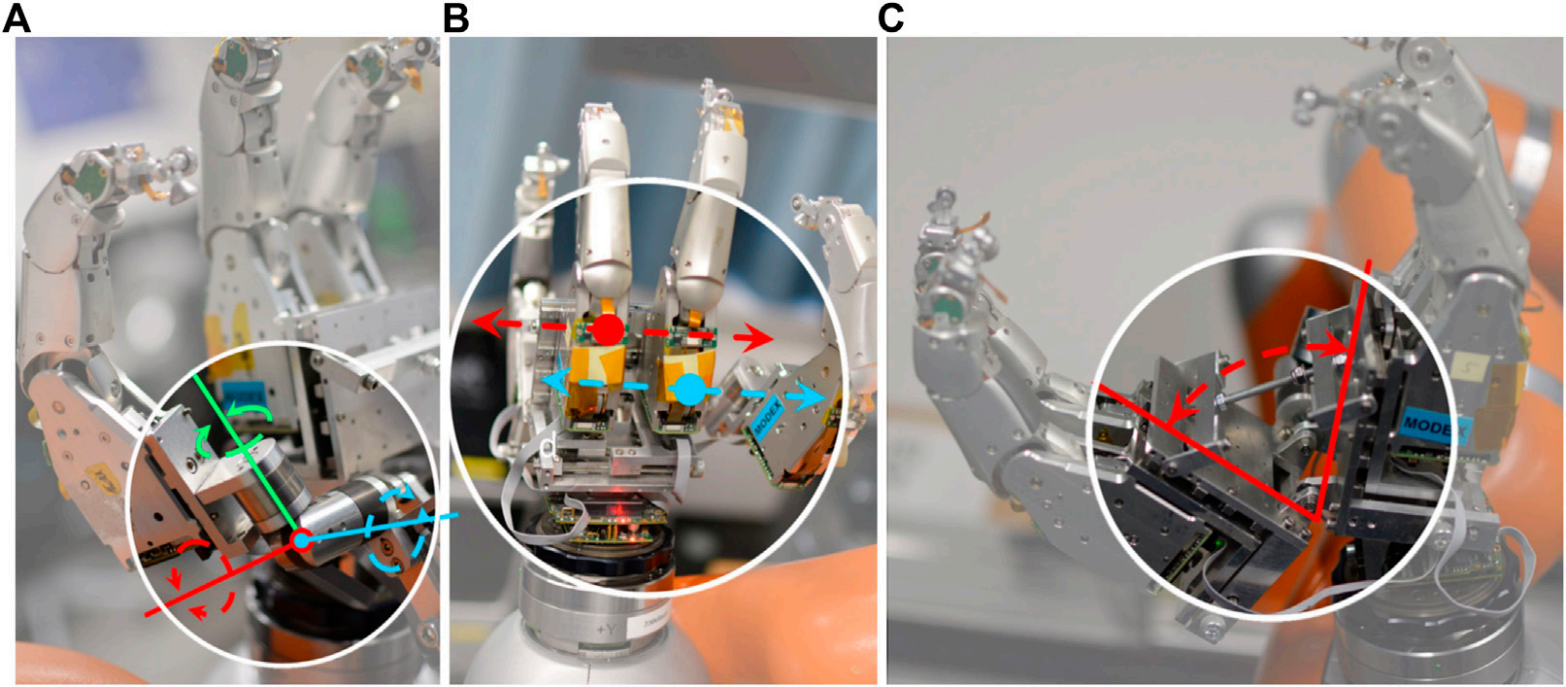
Mechanisms of the eight passive reconfigurable DOFs in the Exodex palm base enabling adjustments to fit different hand sizes. **(A)** Three rotational DOF with friction stop mechanisms. **(B)** Sliding mechanisms for the adjustment of Exodex fingers’ linear positions, which allows for accommodation of different user finger spread distance, as well as palm widths. A total of four such mechanisms are implemented, with two for the user’s index and middle fingers, as well as two for the palm. **(C)** The cupping angle to adjust the palm size, which allows for accommodating different user palm lengths. These adjustment mechanisms also enable the workspace to be tailored to different tasks, such as more open-palm and power grasp of larger objects, or dexterous in-hand manipulation that utilizes the user’s fingertips more.

To realize this, the Exodex palm base is designed with a total of eight reconfigurable DOF. These include one palm cupping angle, four translational positioning, and three rotational DOF for the human thumb’s interacting robot finger. These adjustment mechanisms for reconfiguring the palm base are shown in Figure 4.

Including passive DOF while increasing flexibility of the design in terms of optimal placement of the robotic fingers and hence manipulability during operation also comes with design challenges. Mechanisms take up space and also compromise the mechanical stiffness of the device—the entire device becomes less rigid. Furthermore, the passive DOF are envisioned to be automated in future designs. However, this will introduce further challenges for packaging and increased system complexity.

In the current design, four individual linear adjustments (see Figure 4B) are provided for four of the robotic fingers (two connected to the user’s palm, one to the index finger, and one to the middle finger). They are constructed on high precision, low friction linear bearings with an unobtrusive profile. Quick release screw-type brakes keep the robotic fingers in their desired positions. The linear DOF are particularly suited to address the different hand widths, as they allow for the adjustment to suit the spacing between the user’s fingers, as well as the palm width. The cupping angle (see Figure 4C) of the Exodex palm base can also be adjusted to accommodate different lengths of the user’s hand. As the thumb is the most important member of the hand (Chalon et al., 2010), it is also given the most adjustment DOF in our design to better accommodate different users and use cases. Three rotational joints are incorporated to the base of the robotic finger (see Figure 4A) for pose and position adjustments.

Finally, the palm base also houses the communication gateway and power source to manage the data transition and power supply for the robotic fingers.

### 3.4 Gimbal Joint: Enabling Free Rotational Motion While the User is Connected to the System

To allow free movement of the user’s hand while being attached to the haptic UI, free rotation at the attachment points is required. In Endo et al. (2011), a magnetic ball-in-socket joint is used to allow omnidirectional rotation. The human is attached to a magnetic ball, which fits into a hemispherical socket on the robot side.

An alternative solution is a triaxial gimbal, which has been proposed for force reflection haptic devices (Massie and Salisbury, 1997). A gimbal with ball bearings allows nearly frictionless movement, whereas a ball-in-socket joint can have higher friction at the contact surface. Furthermore, a gimbal mechanism can provide significantly more range of motion.

**FIGURE 5:**
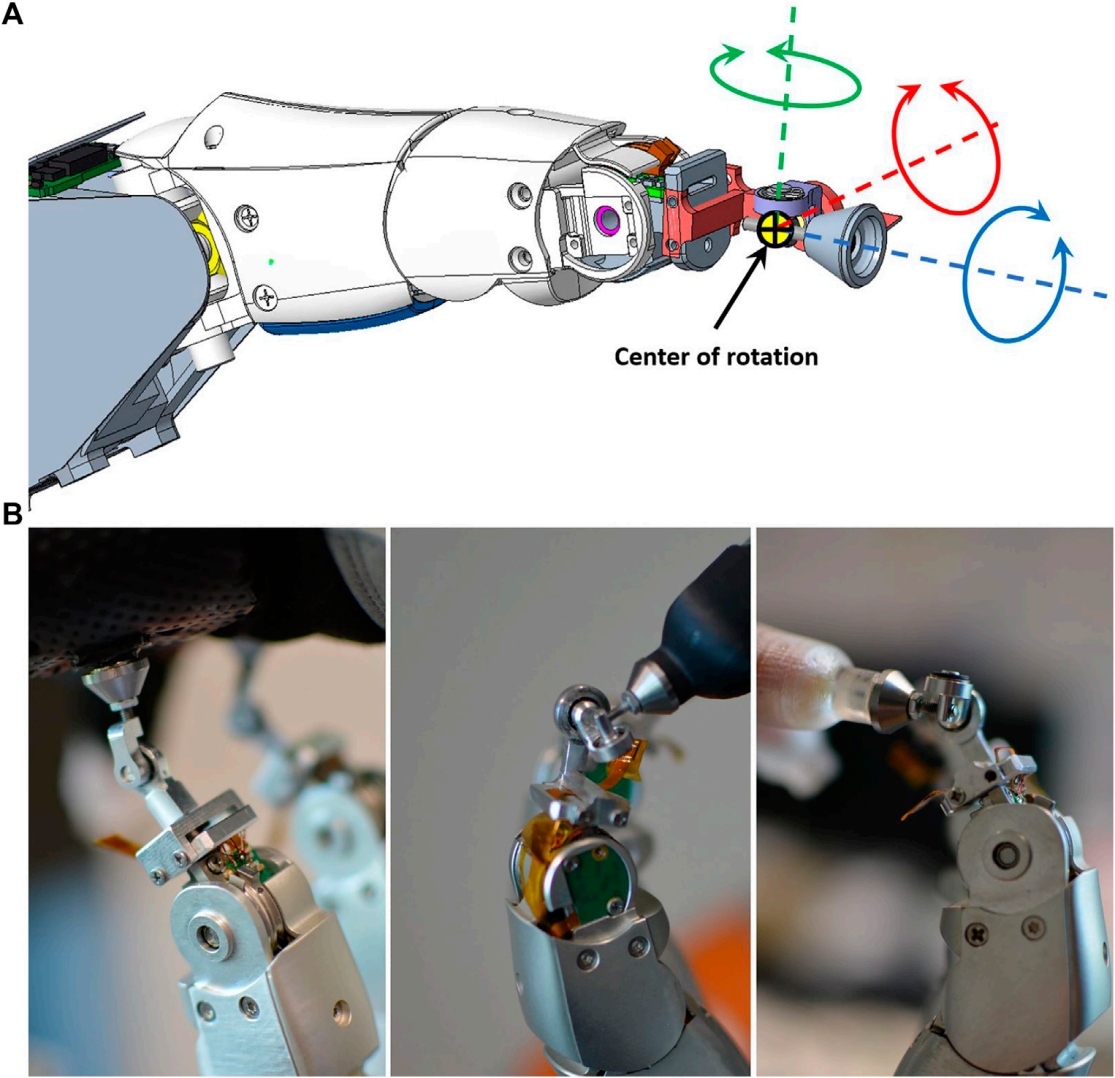
Gimbal rotational DOF and different gimbal arrangements as implemented on the Exodex. The three rotational directions of our gimbal design can rotate 180 deg (green axis), 250 deg (red axis), and endlessly (blue axis). Thanks to low friction bearings, the friction is negligible. **(A)** Three rotational DOF go through a single center point. **(B)** Different mechanisms are needed to avoid gimbal lock on the robotic fingers connected to the palm (left) and those connected to the fingers and thumb (centre, right).

Our design, as shown in Figure 5, employs three rotational axes going through a single center of rotation, which provides the point of reference for the user’s attachment to the system. The three rotational directions of our gimbal design can rotate 180 deg (see Figure 5, green axis), 250 deg (see Figure 5, red axis), and endlessly (see Figure 5, blue axis) in three rotational DOF. This compares favorably against commercial ball-and-socket joints typically capable of a pivot angle of about 40 deg at most (Igus, 2020).

In addition, a gimbal mechanism allows straightforward inclusion of decoupled sensors to measure position or torque in each rotational DOF. This would allow the orientation of the attachment points of the user with respect to the UI system to be measured. This is, however, not yet implemented in the current version of Exodex.

A drawback of gimbals is that they can be subject to gimbal lock. This situation is a singularity which occurs when the first and third axes of the gimbal are aligned. We therefore developed different gimbal configurations as shown in Figure 5. For attachments to the user’s palm, the first axis of the gimbal is parallel to the long axis of the robotic finger’s distal link. For the attachment to the user’s fingers and thumb, the first axis of the gimbal is parallel to the last joint axis of the robot finger. With these different axis-arrangements, we could avoid the occurrence of gimbal lock within the range of motion of the human hand while being attached to the Exodex.

### 3.5 Connecting the User to Exodex

**FIGURE 6:**
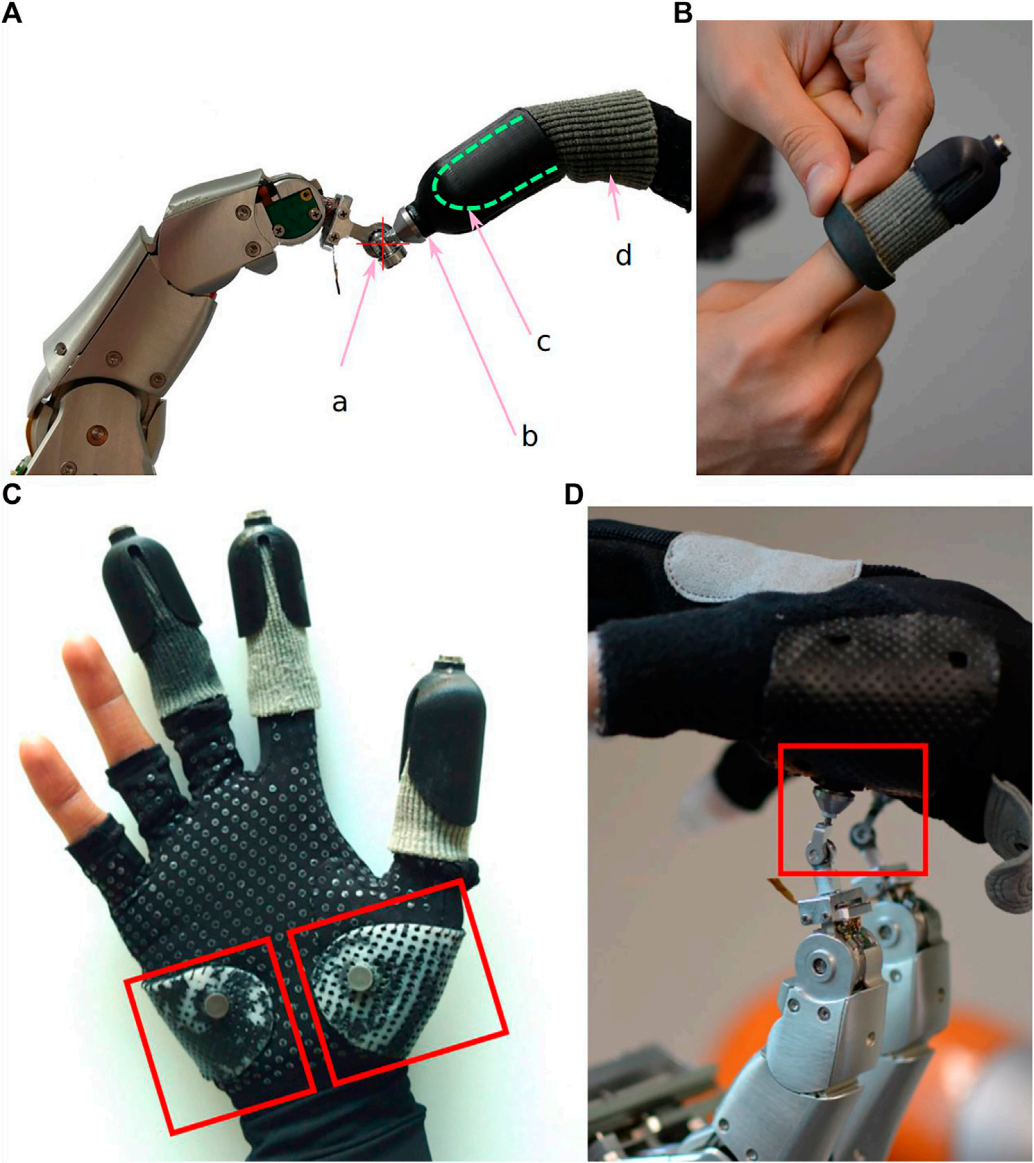
User attachments to the Exodex. **(A)** The 3-DOF gimbal (a) is integrated at the tip of the robotic finger. The magnetic clutch (b) is attached to the gimbal for a rigid connection from the distal phalanx of the user. (c) The positions of the gimbals’ centers are found from forward kinematics of the Exodex. The elastic sleeve is shown in (d). **(B)** Close up of the elastic cloth–silicone attachment: the elastic cloth sleeve is flipped back to show the black silicone lining. **(C)** Open-finger glove with palm attachment points. The attachments are marked in the red boxes. **(C)** The glove as worn by a user. A magnet is integrated into each attachment point on a supporting plate, which allows the forces to be distributed over an area on the palm. **(D)** The user’s hand with the glove attached to the Exodex.

The user’s hand is attached to the Exodex via a magnetic clutch connected to the aforementioned gimbal mounted on the robot fingers. The index and middle fingers and the thumb of the user are fitted into rigid plastic thimbles with a fixed-pose magnetic clutch at the fingertip, as shown in the left side of Figure 6. An elastic sleeve lined with silicone ensures a snug but comfortable fit, while eliminating slippage and reducing play. The transformation from the user’s distal phalanx to the intersection of the gimbal’s axes is therefore constant. The rigidity allows the rendered forces to be transmitted crisply (see Figures 6B,D). We have carried out tests with three different sizes of thimbles, which so far could accommodate all finger sizes that we have encountered.

Magnetic clutches are also placed on the palm on two form-fitting plastic plates on an open-finger glove worn by the user, as shown in Figures 6C,D. These provide the rigidity necessary to transmit forces crisply to the user’s palm surface. This also allows the rendering of a more intuitive force reflection to an area of the palm, rather than feeling like being poked.

To ensure safety, the magnetic clutches detach automatically should a dangerously high force be exerted. The user can simply pull back and away from the haptic UI to safety at anytime. The magnet positions are adjustable to regulate the coupling force according to user and target application requirements. An additional dead man’s switch to automatically bring the system into a safe mode (e.g., compliant mode or full shut down) is being considered to provide additional safety.

An additional benefit of a magnetic clutch is the improvement in the ease of usage for the user to clutch into the Exodex. As the magnets on the user’s hand attract the clutch holders on robotic fingers, they conveniently snap into position when the hand is close by, which makes clutching quite easy for the user.

### 3.6 Extending Exodex Workspace With a Dexterous Robot Arm

The Exodex can function as a stand-alone UI for the hand, particularly in locations with limited space such as inside a spacecraft or research submarine. It is already capable of complex in-hand gesture and manipulation commands, as discussed in Section 7. However, it also limits the workspace of the system for the user. The use of a robotic arm as a haptic UI has been introduced in a recent work on the applications in user arm manipulation (Hulin et al., 2011) and reconfigurable vehicle UI console (Lii and Neves, 2021). We extend upon this approach by integrating the arm as a component of the haptic UI to form a hand–arm UI system.

Figure 1 (at the beginning of Section 3) shows the Exodex with the integrated 7-DOF robotic arm. Specifically, we integrated a DLR-KUKA LWR arm into the Exodex. With seven BLDC motor–driven DOF and joint torque sensing at each joint, as well as a possible additional 6-DOF force–torque sensor at the tool center point (TCP), the robotic arm can truly extend the user’s workspace. With such an extension, the user can employ their arm to explore the environment. It also allows a larger range of motion for the user. The integration of the dexterous arm provides gravity compensation to relieve the user of carrying the weight of the system. It also allows for force reflection to be transmitted to the user’s arm, thus completing an immersive hand–arm haptic UI experience.

## 4 User Attachment Point Placements for Fitting Exodex’s Workspace

To make the best use of the robotic fingers’ workspace and match the desired movement of the human hand, the robotic fingers must be placed such that they allow maximum manipulability in the applications for which Exodex was envisioned. To help enable an effective whole-hand haptic experience, this section examines some of the key workspace considerations to finding suitable attachment configurations, including the positioning of the 3-DOF gimbal, pose and positioning of the robotic finger to the user’s thumb, and effective attachment points on the user’s palm.

### 4.1 Gimbal Placement Considerations for User Workspace

**FIGURE 7:**
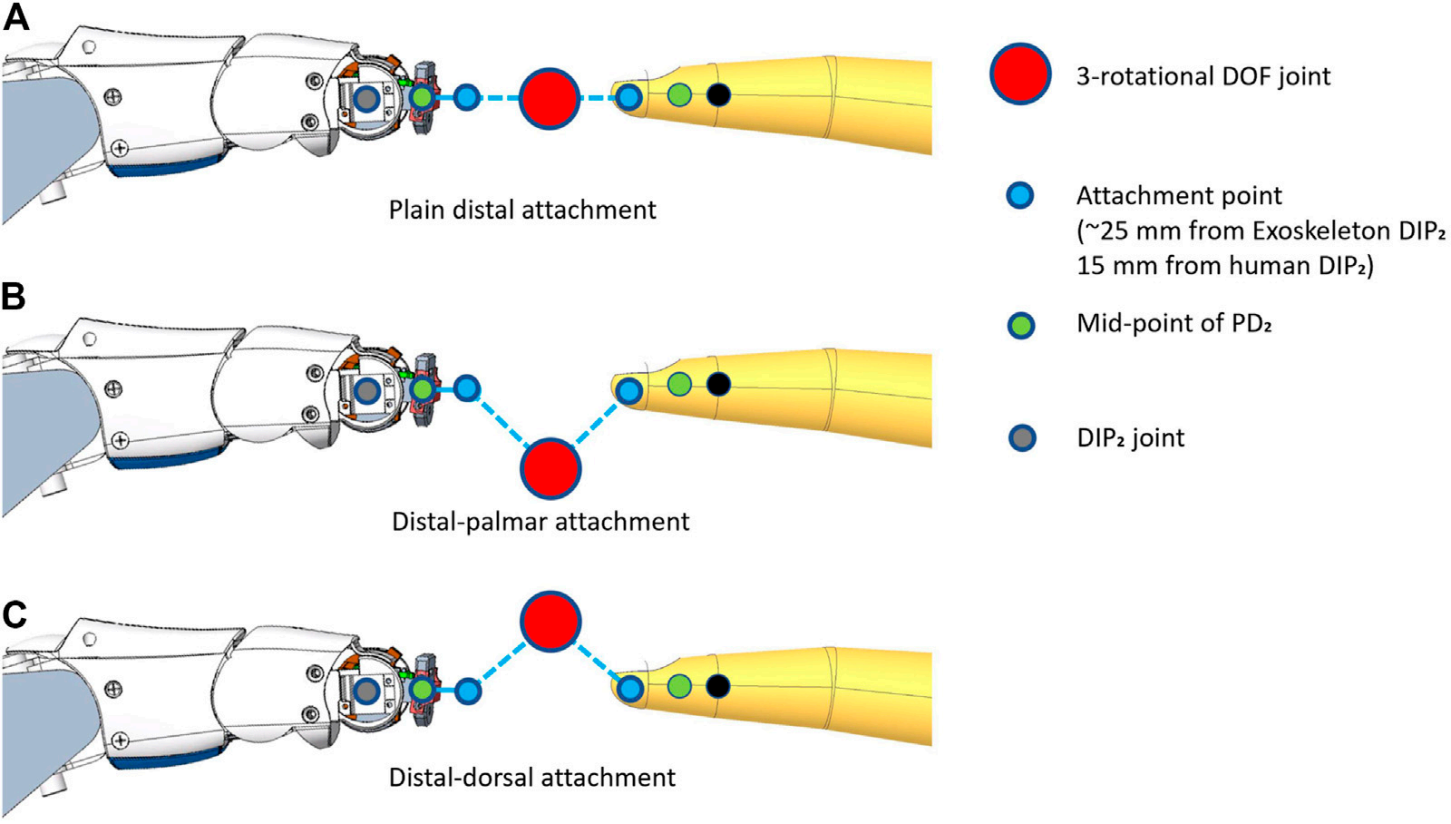
Possible locations for the center of the attachment gimbal. Three different positions are shown here with the attachment: a. in-line with **(A)**, b. below **(B)**, and c. above **(C)** the distal links of the robotic fingers.

**FIGURE 8:**
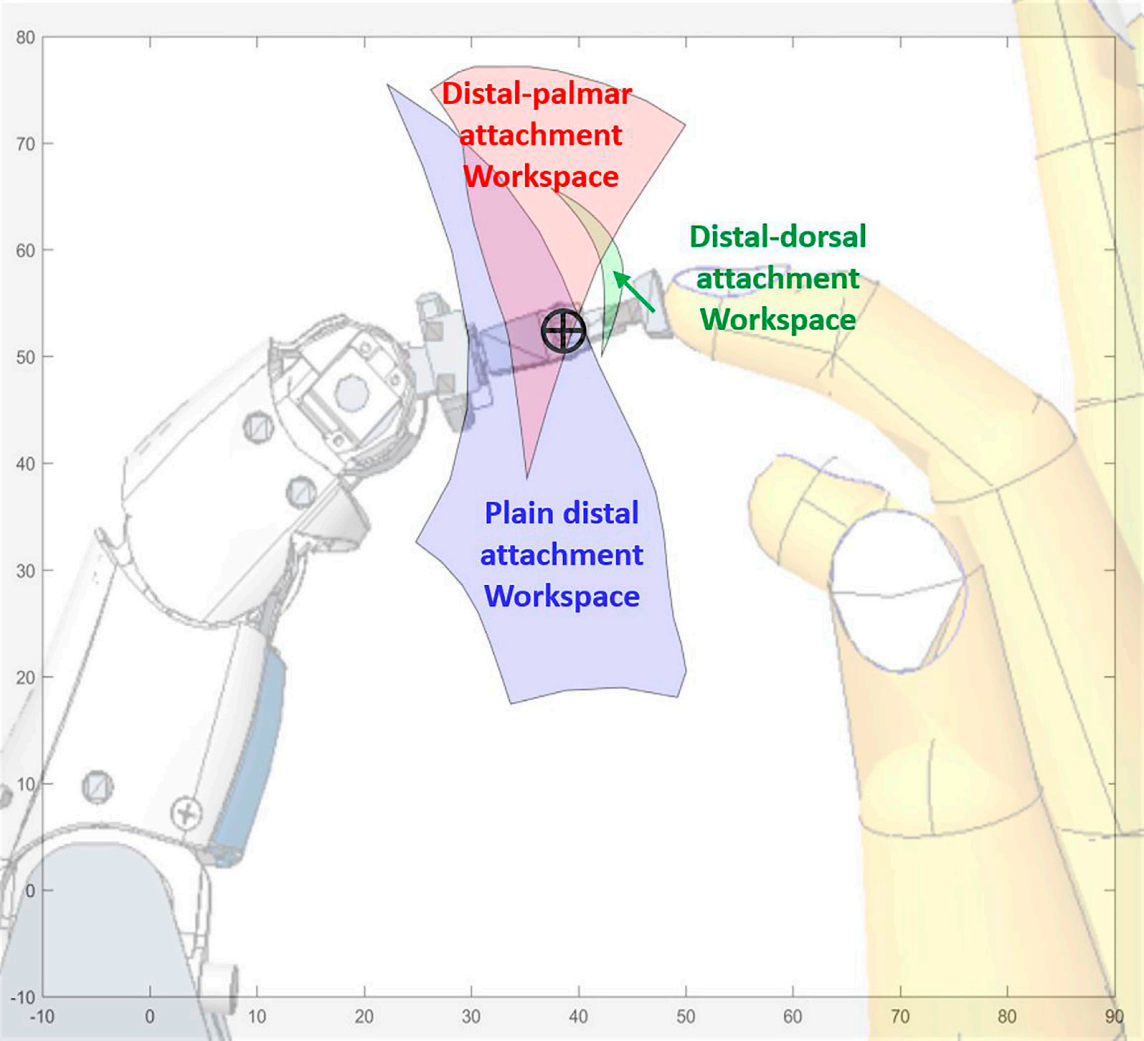
Workspace boundaries of the gimbal center in three different attachment configurations. An overlay of the Exodex finger attached to the user’s index finger is included for clarity. Blue: plain distal attachment. Red: distal-palmar attachment. Green: distal-dorsal attachment.

To ascertain a desirable point for attachment for the user’s index and middle fingers to the robotic fingers, we examined the available workspace for three planar locations: plain distal, distal-palmar, and distal-dorsal. Figure 7 gives an illustration of these attachment positions. Their respective workspaces are visualized in Figure 8, with the robotic finger and a user’s hand superimposed for reference.

The workspace of the plain distal attachment provides the largest available planar workspace at 755.24 mm^2^. The workspace using the distal-palmar attachment measured 392.50 mm^2^ (51.97% of the plain distal workspace). Finally, the distal-dorsal attachment configuration only allowed a very thin, crescent-shaped workspace measuring 20.82 mm^2^ (2.76% of the plain distal workspace). The significantly larger workspace achieved by the plain distal attachment configuration is therefore implemented in the Exodex.

### 4.2 Examining the User’s Thumb Workspace

**FIGURE 9:**
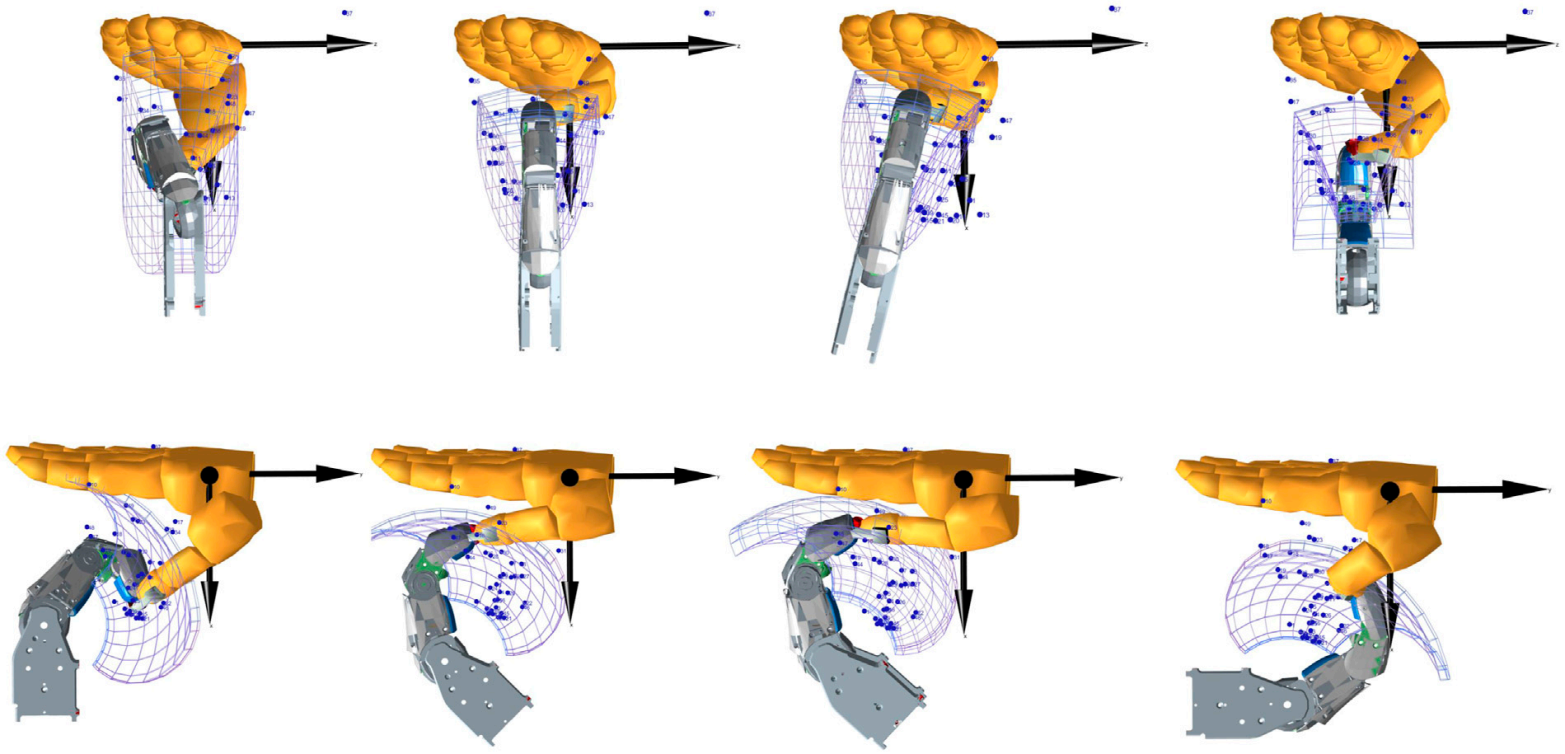
Different options of placing the robot finger base with respect to the palm that allow good workspace of the thumb for in-hand manipulation. Top: views from the fingertip side (dorsal views). Bottom: views from the thumb side (radial views).

The thumb and its omnidirectional range of motion is essential for dexterous (in-hand) tasks. Consequently, we implemented the most reconfigurability for its corresponding attachment with three adjustable joints. To examine the suitable workspace that these pose adjustments can provide for the user, we explored different attachment configurations of the user’s thumb by examining the possible workspace with the user’s thumb attached to the robotic finger in different base configurations. This is made possible utilizing various hand poses taken from magnetic resonance imaging (MRI) measurements (Stillfried et al., 2014). Examples of their workspace with a variety of base positions and orientations of the robot finger are visualized in Figure 9. The center position of the gimbal joint with the thumb connected to the robotic finger is calculated for each pose, as denoted by the blue dots. Furthermore, the workspace of the center of the gimbal joint on the TCP of the robotic finger is plotted as a thin line mesh. The base of the robot finger is moved until there is a good congruence between the gimbal center positions from the MRI measurements and the gimbal range of motion from the robot finger.

We observe in Figure 9 that the workspace of the robotic finger becomes long and thin as the finger becomes extended. However, full extension is a singularity, and near full extension, manipulability is low. As such, this part of the workspace is limited in usefulness. To avoid bringing the robotic fingers into full extension, the palm of the Exodex is designed in such a way that the distance between the opposing robotic fingers can be adjusted using the adjustable cupping angle. This way, depending on the size of the operator’s hand, the cupping can be adjusted so that the robotic finger is always in at least a slightly flexed posture.

As expected, the entire range of motion of the human thumb cannot be covered with the robotic finger’s available workspace. A further observation is that all different poses achieve similar workspace volumes. This is particularly noticeable from the dorsal view in Figure 9. This is a result of the human thumb’s vastly larger reachable workspace as compared to the robotic finger. Conversely, we can conclude that the human thumb can exploit the full workspace of the robotic finger. Nevertheless, the achieved workspace would already allow good performance by matching the palm base configuration to the desired tasks, such as gesture command, or in-hand dexterous manipulation. This is further confirmed in several use cases, which are detailed in Section 7. This also confirms our design strategy of implementing a reconfigurable base to compensate for the limitation of the robotic fingers available.

An interesting, albeit failed, attempt was made to increase the workspace for the user’s thumb by increasing the length of the distal link of the robotic finger connected to the thumb tip. However, this led to instability in the form of vibration during testing. This appeared to be caused by the flexibility in the mechanism for actuating the distal phalanx of the robotic finger. Specifically, this may stem from the cable-driven coupling between the distal and medial links, combined with the flexibility from the aluminum extension to the distal phalanx. Setting the finger feed-forward gains (see Section 6.4) lower in turn resulted in a large perceived inertia, which made it difficult for the user to move the thumb when attached to the Exodex.

### 4.3 Providing Whole-Hand Immersion Through Palm Attachment

**FIGURE 10:**
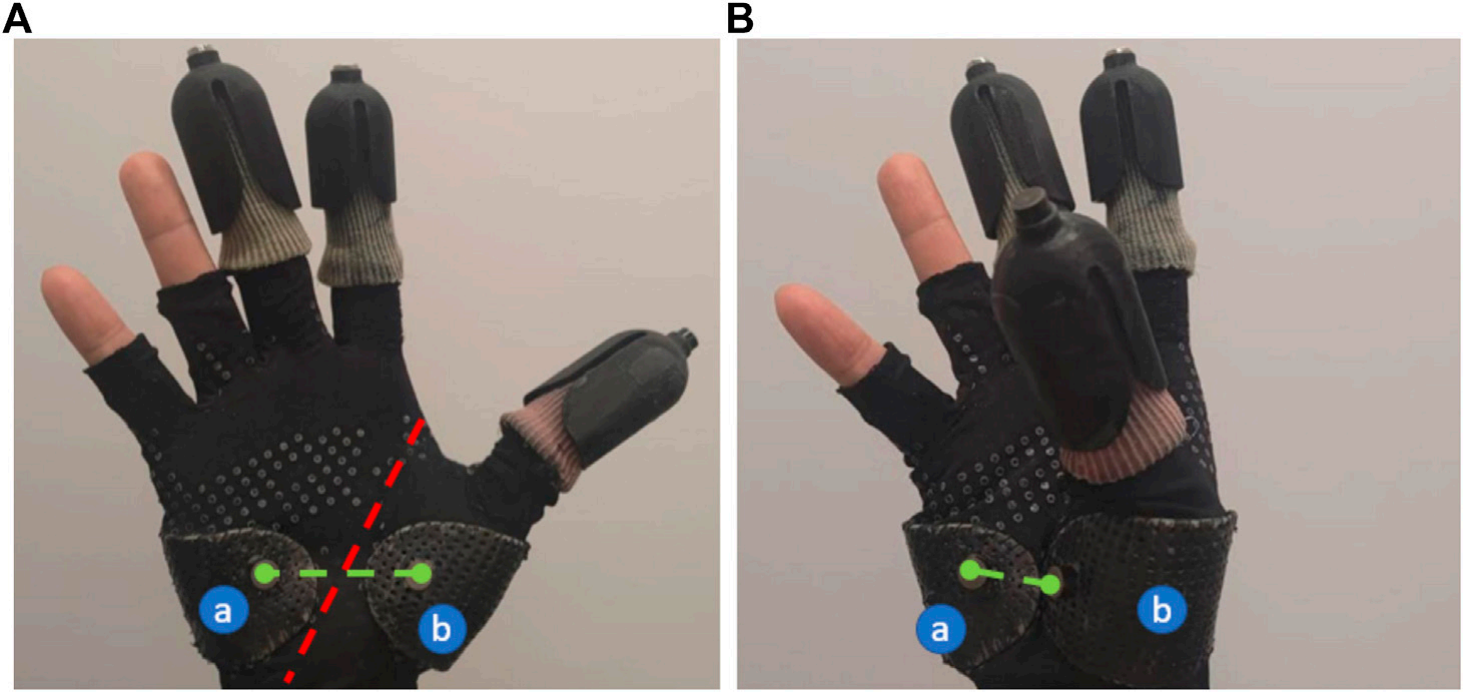
Demonstration of the palm motion that can be captured by the Exodex. The attachments on the support plates covering the thenar bulge (a) and the hypothenar bulge (b) transmit the forces and motion between the user and the Exodex. The red dashed line notes the axis of the circumduction/folding motion of the palm. By placing the two attachments points on either side of this line, this motion can be captured by the Exodex. The difference in the green dashed line between the **(A,B)** figures gives an impression of the movement possible by the user’s palm.

As already discussed earlier, the incorporation of palm interaction enables the Exodex to provide a whole-hand interaction, as well as to estimate the pose and gesture. Two attachment points have been implemented on the user’s palm, as shown in Figure 10. With sparser touch receptors in the palm than at the fingertips, we expect that one attachment on each side of the palm would provide sufficient haptic feedback to the user. The support plates allow the reflected forces to be distributed more evenly over the palm surface.

For pose estimation, the placement of the attachment points on the thenar bulge (thumb side) and hypothenar (little finger side) also enables the two robotic fingers to capture the pose of the palm, as well as the circumduction folding angle, which in turn helps estimate the whole-hand gesture. Furthermore, as the thenar bulge is directly connected to thumb metacarpal link, it is also helpful in estimating the thumb’s pose relative to the whole hand. The effectiveness of this setup is particularly evident in the gesture recognition experiments as discussed later in Section 7.2.

## 5 Optimization of Device Kinematics

Given the inherent workspace and collision limitations of a front-facing haptic UI or hand exoskeleton, it is impossible to achieve the entire range of movement of a healthy human hand while connected to the Exodex, since 1) the attachment points are at the fingertips, meaning it is impossible to, e.g., ball the hand into a tight fist without detaching the fingers, and 2) due to the limited range of motion of the robot fingers compared to that of the human fingers and the need to avoid collisions between robot fingers (e.g., one cannot cross one’s fingers while connected to the Exodex).

Nevertheless, some key hand configurations required to explore or manipulate a real or virtual environment should be achievable, and manipulability of the system should be maximized for a hand configuration (or configurations) relevant to manipulation. As shown in Figure 4, the Exodex has eight passive DOF to accommodate users with various hand sizes and shapes.

In this section, we show how we set the positions of the eight passive DOFs shown in Figure 4 and described in Section 3.3 to optimize the workspace for different users. The optimization is high-dimensional, since there are not only the eight passive DOF ***y*** of the palm base, which can be optimized for any given hand pose, but also the transformation ***q***_pose_ between the human hand’s base coordinate system and the base coordinate system of the Exodex is not fixed.

We use a two-step, iterative approach to tackle this problem. Starting with an initial guess of the positions ***y*** of the passive DOF, the first step (lines 6–9 of **Algorithm 1**) is to determine whether there exists a pose of the hand’s base coordinate system for which the joint angles of the robotic fingers are within limits, for each key hand configuration. The second step (lines 16–19) is to modify the positions ***y*** to increase manipulability of the robotic fingers for the configurations relevant for manipulation. These two steps are iterated until convergence or until a maximum number of iterations. In case, in the first step, there are some configurations for which no pose of the hand base brings the joint angles within limits, we go back to the last feasible ***y***, and perform the second optimization step, i.e., maximizing the manipulability, for these failing configurations instead of the configurations relevant for manipulation. This is shown in lines 10–14.

### 5.1 Manipulability Measure

Central to the optimization algorithm is a manipulability measure, defined per robotic finger, of the contact point with respect to the base of the finger *i* with joint values $\theta_{i}={\left[\theta_{1},\theta_{2},\theta_{3}\right]}^{⊤}$ , shown in Eq. 3. If the contact point is within the workspace of the robotic finger, the manipulability measure is Yoshikawa’s manipulability measure (Yoshikawa, 1985) combined with a penalization function *P*(***θ***_***i***_) for joint limits as described in Tsai (1986, Eq. 84). The parameter *k* regulates the slope near the boundary of the workspace, and *θ*_*j*_, *θ*_min,*j*_, and *θ*_max,*j*_ are the values of the *j*th joint and its lower and upper limits, respectively. Note that if *J* is a square matrix, as it is in our case, then $\sqrt{\text{det}\left(JJ^{⊤}\right)}=\text{det}\left(J\right)$ . If the contact point is outside the robotic finger workspace, then the measure is *ad*(***x***)^2^ + 2*bd*(***x***), where *d* is the distance to the closest point on the finger workspace and *a* > 0 is the gradient at the workspace boundary. We set *k* = 100, *a* = 1, and *b* = .2.

$$\begin{array}{ccc} \left[\theta_{i},inBounds\right]=inverse\text{\_}kinematics\text{\_}finger\left(x_{i}\right) & & \end{array}$$ (1)

$$\begin{array}{ccc} P\left(\theta_{i}\right)=1-\text{exp}\left(-k\overset{3}{\underset{j=1}{\prod }}\frac{\left(\theta_{j}-\theta_{\min,j}\right)\left(\theta_{\max,j}-\theta_{j}\right)}{{\left(\theta_{\max,j}-\theta_{\min,j}\right)}^{2}}\right) & & \end{array}$$ (2)

$$\begin{array}{ccc} m\left(x\right)=\left\{\begin{array}{cc} \text{det}\left(J_{F}\left(\theta_{i}\right)\right)P\left(\theta_{i}\right),\; & \text{if}\;inBounds\\ ad{\left(x\right)}^{2}+2bd\left(x\right),\; & \text{otherwise} \end{array}\right. & & \end{array}$$ (3)

Algorithm 1 Update hand state.

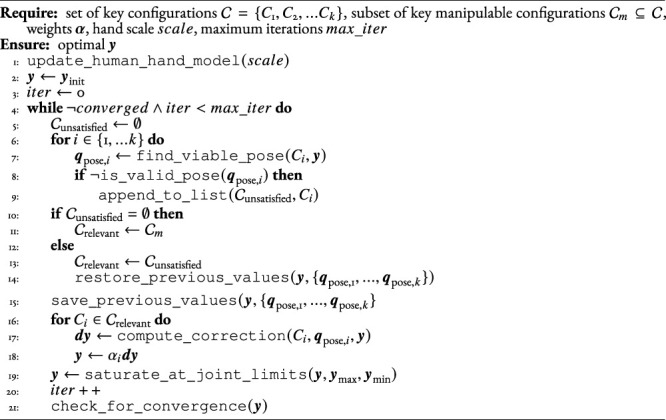

### 5.2 Determining a Valid User Hand Pose

To find a valid pose, we use **Algorithm 2**: find_viable_pose . This first tries gradient descent, and if this fails, tries Particle Swarm Optimization (PSO). PSO (Clerc, 2006) is a sampling-based method that works in the non-convex and non-smooth domains and may find a viable ***q***_pose_ where gradient descent fails.

Our implementation of gradient descent finds, at each step, the gradient of the manipulability index (Eq. 3) at each contact point. It then sums these gradients and multiplies by a gain to yield the change in translation of ***q***_pose_ and sums their cross products with the distances from the hand’s base coordinate system to each contact point, multiplied by another gain, to yield the change in orientation of ***q***_pose_.

Algorithm 2
find_viable_pose

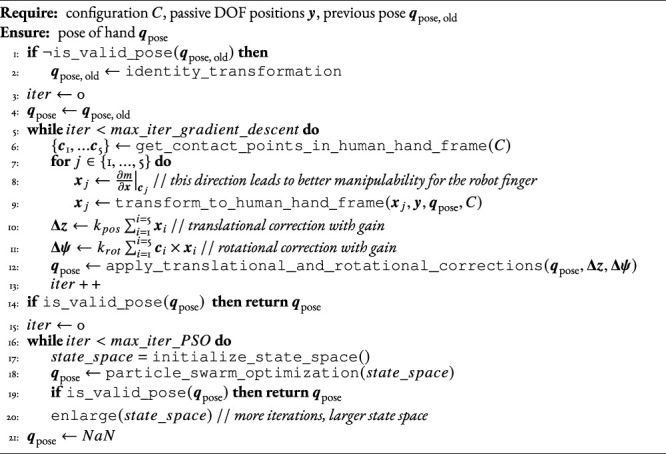

### 5.3 Optimization of Passive Degree of Freedom Using Nullspace Projection

The calculation of the iterative correction ***dy*** in line 17 of **Algorithm 1** is described in **Algorithm 3**. It is found by calculating, for each contact point, the gradient of the manipulability index (Eq. 3) with respect to the joint angles of the robotic finger. These are then multiplied by a gain, and projected into the nullspace of the entire Exodex, i.e., the finger joints and the passive DOF. The nullspace projector projects any vector of forces on the Exodex’s DOF into a subspace: the nullspace. Any force in the nullspace will not produce a resultant force at the contact points.

Letting $x_{i}\in R^{3}$ be the Cartesian position of the *i*th attachment point, then $x={\left[x_{1}^{⊤},x_{2}^{⊤},x_{3}^{⊤},x_{4}^{⊤},x_{5}^{⊤}\right]}^{⊤}$ is the vector of attachment points. Letting $\theta_{i}\in R^{3}$ be the joint positions of the *i*th robotic finger, $\theta ={\left[\theta_{1}^{⊤},\theta_{2}^{⊤},\theta_{3}^{⊤},\theta_{4}^{⊤},\theta_{5}^{⊤}\right]}^{⊤}$ is similarly the vector of the Exodex’s active joint positions (just like ***y*** is the passive DOF positions). Then let ***J***_***θ***_ be the Jacobian of ***x*** with respect to ***θ***, ***J***_***y***_ be the Jacobian with respect to ***y***, and ***J***_***y***, ***θ***_ = [***J***_***y***_, ***J***_***θ***_].

The static nullspace projector ***N*** was calculated as in Dietrich et al. (2015):

$$\begin{array}{ccc} N=I-J^{⊤}{\left(J^{W+}\right)}^{⊤}, & & \end{array}$$ (4)

$$\begin{array}{ccc} J^{W+}=W^{-1}J^{⊤}{\left(JW^{-1}J^{⊤}\right)}^{-1}, & & \end{array}$$ (5)

where ***J*** ^***W*** +^ is the weighted pseudoinverse of the Jacobian using diagonal weighting matrix ***W*** (we omit the dependency on ***q*** for brevity). Depending on ***W***, we can increase or decrease the contribution of various DOF. Since we are interested in the contribution of the passive DOF of the Exodex, we adjust the weighting accordingly, setting the values of ***W*** on the diagonal which correspond to the passive DOF low, and the others, which correspond to pose, high.

Algorithm 3
compute_correction

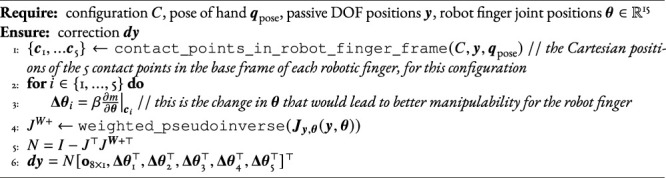

## 6 User State Estimation and Control

For accurate contact rendering and force-feedback from a virtual or remote environment, it is required to know the position and orientation of the hand (hand pose), and the positions of the joint angles in the fingers and the palm (hand configuration).

There are a number of ways to measure these. The configuration could be acquired from strain gauges on a sensorized glove, e.g., Cyberglove. The pose could be found from IMUs placed on the human hand, as long as the device is used in microgravity. These methods require electronics to be attached on the user’s hand. Another possible method is through vision or motion-capture systems to capture pose and finger angles. However, this can be susceptible to occlusion and mislabeling.

In Pereira et al. (2019), we show how to reconstruct the hand pose and configuration only from the positions of the attachment points of the Exodex to the human hand. These can be determined from the angular position sensors on the robot finger joints, and the known kinematics of the system. Inverse kinematics can then be performed on a joint model of the human hand to determine the pose and configuration. The user does not need to wear any sensors.

### 6.1 Hand Model

**FIGURE 11:**
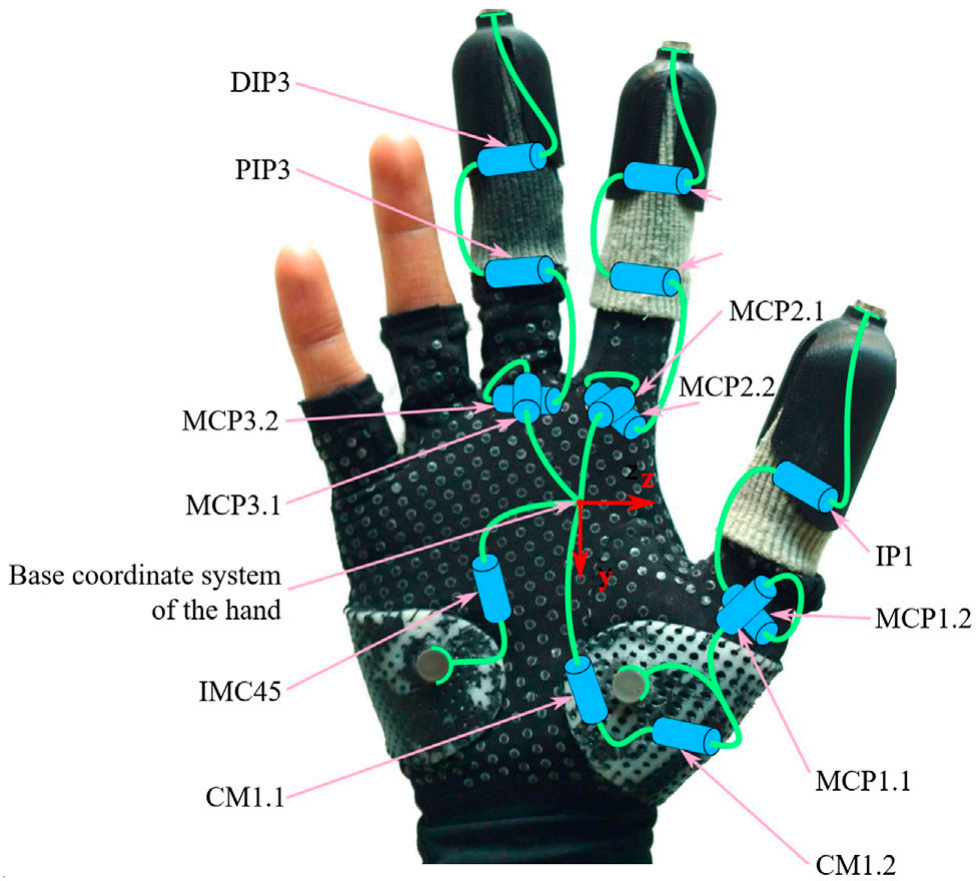
Diagram of hand kinematic model overlaid on human hand wearing glove and finger socks. Cylinders represent revolute joints; green lines represent links. Not shown: three prismatic and revolute joints linking world coordinate system with the hand’s base coordinate system (this shown here in red).

The human hand model for the pose estimation is derived from MRI data and is based on Stillfried et al. (2014). The original model has 22 DOF: five in the thumb, four in each finger, and the intermetacarpal joint in the palm. However, neglecting the human’s last two fingers and setting the distal interphalangeal joint (DIP) and proximal interphalangeal joint (PIP) to be proportional (constant *k* is the ratio DIP:PIP), the number of DOF reduces to 12. Added to this the six DOF in the hand pose (i.e., position and orientation of the hand’s base coordinate system) and there are 18 DOF to be determined from 15 constraints [three-dimensional (3D) positions of each attachment points]. The kinematic model is shown in Figure 11 overlaid on an image of the glove and attachment points.

In Pereira et al. (2019), constant *k* is found to give the most accurate tracking at $k=\frac{1}{2}$ and $k=\frac{2}{3}$ . This corresponds with the value from the empirical measurements in Rijpkema and Girard (1991) and the value of $\frac{1}{2}$ suggested for power grasps in Cobos et al. (2008). We used a value of $k=\frac{2}{3}$ in our trials in Section 7.

### 6.2 Iterative Hand State Update

The well-known iterative method for the inverse kinematics which minimizes the joint error using the transpose of the Jacobian (Balestrino et al., 1984; Wolovich and Elliott, 1984) is used. As there are more DOF than constraints, a nullspace of dimension 18 − 15 = 3 exists. In the nullspace, we optimize away from the joint limits in the hand to achieve a more natural posture. The full algorithm is recapitulated in **Algorithm 4**.

Algorithm 4 Update hand state.

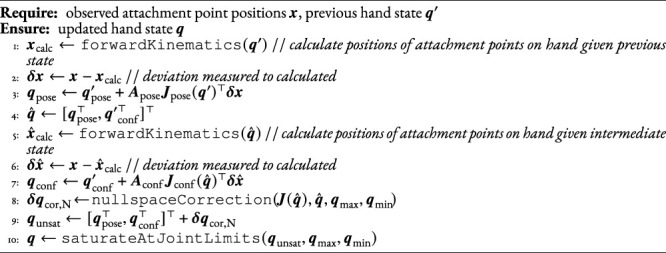

In the algorithm, $q\in R^{3}\times SO\left(3\right)\times R^{12}$ is the 18-dimensional vector of joints of the hand. This consists of $q_{\text{pose}}\in R^{3}\times SO\left(3\right)$ as the pose of the hand (orientation given in roll-pitch-yaw) and $q_{\text{conf}}\in R^{12}$ as the configuration of all the independent DOF in the hand as described in the previous section. The hand state at the previous time step is ***q*** ′, the vector of attachment point positions at the current time step is ***x***, and the partial derivatives matrices of ***x*** with respect to ***q***_pose_ and ***q***_conf_ are ***J***_pose_ and ***J***_conf_, respectively. ***A***_pose_ and ***A***_conf_ are gain matrices.

A two-step approach is applied where first the pose is updated in line 3, the estimated contact points are recalculated, and then the configuration is updated in line 7. This was found during development to allow higher gains ***A***_pose_ and ***A***_conf_ without becoming unstable, and therefore a faster convergence, than if all state elements were updated at once.

The joint ranges are enforced in line 10; the values are taken from González Camarero et al. (2015), with the exception of PIP2 and PIP3, which are limited in extension to .03 rad. The reason for disallowing full extension and hyperextension was that the restoring values when moving back into flexion, $A_{\text{conf}}J_{\text{conf}}\left(\hat{q}\right)\delta x$ in line 7 would be zero at full extension (since this is a singularity). In hyperextension, these would instead try to pull the joints further into extension.

### 6.3 Nullspace Projection

To move joints away from their limits where possible, which also results in a more natural-looking reconstruction, a correction in the nullspace was defined. This is shown in lines 8 and 9. The nullspace projector was calculated with the intermediate values $\hat{q}$ , to avoid recalculating ***J***_conf_, since this is computationally expensive.

For each DOF, a correction was calculated so:

$${\delta q}_{i,cor}=\alpha_{i}\frac{q_{i,\max}+q_{i,\min}-2q_{i}}{q_{i,\max}-q_{i,\min}},$$ (6)

where for the *i*th DOF, *α*_*i*_ is a gain; *q*_*i*_, *q*_*i*, max_, *q*_*i*, min_, and *δq*_*i*,cor_ are the *i*th elements of the vectors ***q***, the joint limits ***q***_max_ and ***q***_min_, and ***δq***_cor_, respectively. We then project this into the nullspace: ***δq***_cor, N_ = ***Nδq***_cor_, and add it to the hand state in line 9.

The nullspace projector is calculated as in Eq. 4. We omit the dependency on ***q*** for brevity. Since the unweighted pseudoinverse (i.e., with ***W*** = ***I***) can be calculated using singular value decomposition (SVD) (Golub and van Loan, 2013) in polynomial time, we rearrange the Jacobian, defining:

$$W^{-1}=\Omega \Omega^{⊤},\;H=J\Omega ,$$ (7)

Hence Eq. 5 becomes:

$$J^{W+}=\Omega H^{⊤}{\left(HH^{⊤}\right)}^{-1}=\Omega H^{I+},$$ (8)

which can be solved using SVD. Our weighting matrix was a diagonal matrix with low weights for the elements of the pose and the intermetacarpal joint, and high weights for most other joints, as in Pereira et al. (2019). The nullspace projector was calculated in parallel with the robot control at 1 kHz, since the computation was too intensive to be done in serial in a single control cycle.

### 6.4 Control of the Hand–Arm System

**FIGURE 12:**
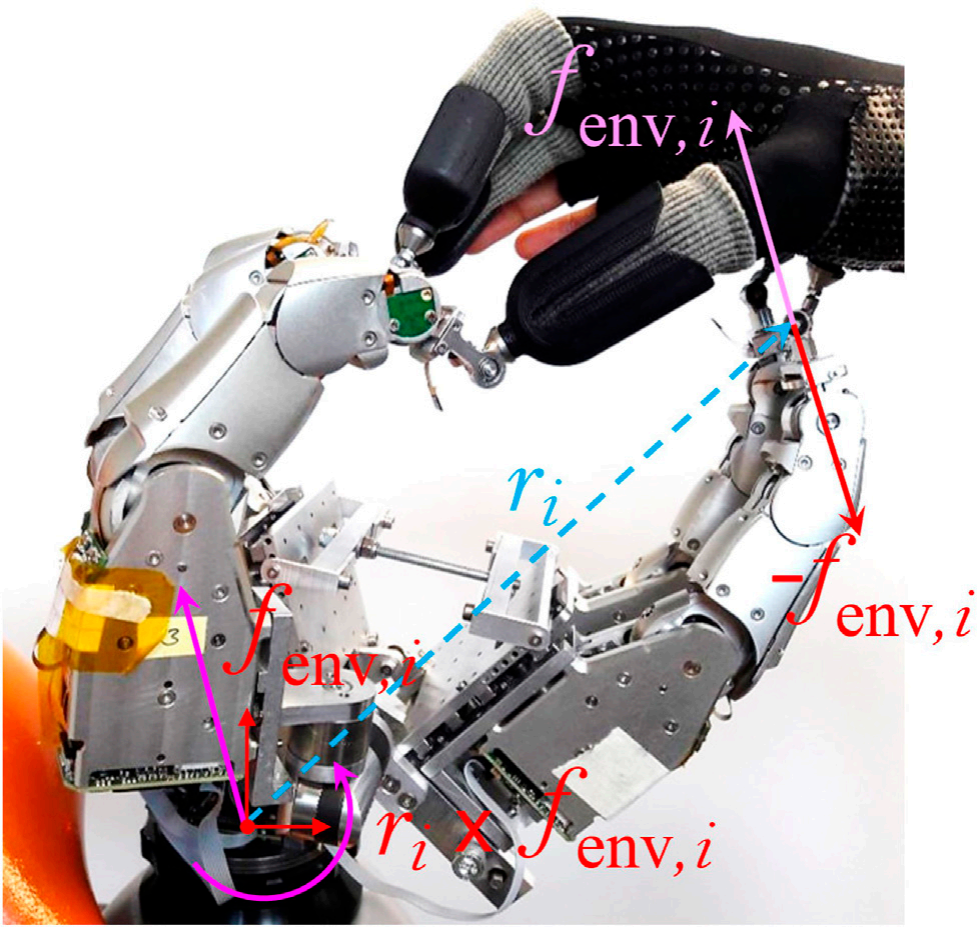
The wrench at the Exodex base (attachment to tool center point of the Light Weight Robot) from an external force at the attachment point to the human. The freely rotating gimbals at the attachment points mean that torques from the human are not transmitted, only forces.

When used in combination with the Exodex, the LWR is controlled in torque mode, compensating only for its weight and compliant to external torques. Attaching the Exodex to the end effector and compensating for its weight means that the Exodex behaves as a floating object in zero-gravity, except that the inertia of the system and a small amount of friction is felt by the user (Schmidt et al., 2020). Forces exerted by the robotic fingers of the Exodex on the human hand leads to a movement of the base of the Exodex if not also counteracted by the LWR (see Figure 12). For this reason, forces from the virtual or remote environment are also applied on the base of the robot similar to that in Endo et al. (2011).

Each of the five robotic fingers are also compliant to external forces and torques, and are gravity-compensated. However, the non-backdrivability of the robotic fingers, as well as their high friction and inertia, mean that feed-forward control and friction compensation are necessary to bring the user closer to an impression of moving in free space. Measured torques on each of the three joints of each finger $\tau_{\text{msr, exo}}\in R^{15}$ are fed forward with a feed-forward diagonal gain matrix *K*_FF_, which lowers the perceived inertia in the mechanism. Gravity compensation $\tau_{\text{g, exo}}\in R^{15}$ is added. The forces from the virtual environment on each finger $f_{\text{env, exo}}={\left[f_{\text{env},1}^{⊤},f_{\text{env},5}^{⊤}\right]}^{⊤}$ , where ***f***_env,*i*_ is the force from the environment on the *i*th robotic finger, are transformed into the joint space using the transpose of the Jacobian $J_{exo}^{⊤}$ , which relates the Cartesian velocity of the contact points in the Exodex base frame to the velocity of each joint. Friction compensation as in Le Tien et al. (2008) is performed on the resulting commanded torque.

$$\begin{array}{ccc} \tau_{\text{des, exo}}=K_{\text{FF}}\tau_{\text{msr, exo}}+\tau_{\text{g, exo}}+K_{\text{FF}}J_{exo}^{⊤}f_{\text{env, exo}} & & \end{array}$$ (9)

$$\begin{array}{ccc} \tau_{\text{cmd, exo}}=\tau_{\text{des, exo}}+\tau_{\text{fric, exo}}\left(\tau_{\text{des, exo}},\tau_{\text{msr, exo}},\dot{q}\right) & & \end{array}$$ (10)

where ***f***_env,*i*_ is the desired force-feedback from the virtual environment. The Exodex base frame is coincident with the end effector frame of the LWR. The control of the LWR is therefore:

$$\begin{array}{ccc} w_{\text{env}}=\underset{i\in \left\{1,\ldots ,5\right\}}{\sum }\left[\begin{array}{c} f_{\text{env},i}\\ r_{i}\times f_{\text{env},i} \end{array}\right] & & \end{array}$$ (11)

$$\begin{array}{ccc} \tau_{\text{LWR,cmd}}=\tau_{\text{LWR,g}}+J_{LWR}^{E⊤}w_{\text{env}}+\left(\tau_{\text{ff}}\right) & & \end{array}$$ (12)

The term ***τ***_ff_ is optional feed-forward term for reducing inertia in free space and is detailed in the next section. Note, as it is only used in free space, the wrench from the environmental forces on the Exodex ***w***_env_ will be zero, and this term does not need to be scaled with the feed-forward gains.

### 6.5 Inertia Reduction in Free Space

Since forces on the LWR and the Exodex are not measured at the end effectors but only in the joints, the movement in free space is not without some perceivable resistance (see Section 7.4). To attempt to remove this resistance, when working in free space, an extra feed-forward wrench on the end effector of the LWR can be introduced. However, using the torques measured at the LWR joints themselves can be inaccurate. As there is a chain of mechanisms between the human hand and the LWR joints (e.g., magnetic clutch attachments, gimbals, Exodex fingers, modular palm, etc.), each component in this mechanism chain can introduce some element of elasticity and play. Therefore, in order to capture the forces that the human exerts as accurately as possible, we measure them as close as possible to the point where they are exerted on the Exodex’s fingers. More accurate would be to measure the forces directly exerted by the human hand on the Exodex. To do this, the forces exerted at the attachment points to the robotic fingers, ***f***_ext,*i*_, are calculated from the torques on the robotic finger joints and are used to determine the total force and torque that the user exerts on the Exodex.

The world frame of the human hand is the same as that of the LWR. The transformation $T_{0}^{H}$ is the transformation from the world to the human hand coordinate system (i.e., applied to coordinates of a point in the hand frame, yielding the coordinates in the world frame) obtained from the first six elements of the hand state ***q***_*pose*_. $T_{0}^{E}$ is the transformation from the world to the end effector. $T_{H}^{E}={\left(T_{0}^{H}\right)}^{-1}T_{0}^{E}$ is therefore the transformation from human hand to end effector.

The torque around the hand’s base coordinate system is also found, as shown in Figure 12. This is the wrench exerted on the robot measured at the base coordinate system of the human hand,

$$w_{\text{ext, H}}=\underset{i\in \left\{1,\ldots ,5\right\}}{\sum }\left[\begin{array}{c} f_{\text{ext},i}\\ \left(T_{H}^{E}r_{i}\right)\times f_{\text{ext},i} \end{array}\right],$$ (13)

where ***r***_*i*_ is the finger contact in the frame of the LWR end effector as in Eq. 11 and shown in Figure 12. Hence, Eq. 13 gives the wrench around the human hand coordinate system in the hand frame. This is translated to the desired LWR joint torques using the transpose of the Jacobian $J_{LWR}^{H}$ of the forward kinematics of the human hand’s base coordinate system. This is calculated from the Jacobian at the end effector $J_{LWR}^{E}$ and the adjunct of the transformation from the end effector to hand base. The gain *K*_*FF*,*free*_ is a diagonal matrix regulating the strength of the feed-forward.

$$J_{LWR}^{H}=\text{adj}\left(T_{H}^{E}\right)J_{LWR}^{E}$$ (14)

$$\tau_{\text{ff}}=K_{FF,free}J_{LWR}^{H⊤}w_{\text{ext, h}}$$ (15)

## 7 Exodex Deployment and Evaluation

To examine Exodex’s ability to effectively serve as a hand haptic UI in a variety of use cases and command modalities, we deployed it in three different scenarios. We first discuss its use in a virtual environment for interacting with mixed media of both solid objects of different shapes and liquids of different viscosities. The second use case realizes hand gesture recognition and telecommand of an avatar robot, for a variety of hand sizes. Thirdly, we present hand–arm teleoperation utilizing adaptive MMT.

### 7.1 Whole-Hand Perception of Liquid and Solid in a Virtual Environment

A virtual environment was created with a cylinder, sphere, and flat surface. The surface object could be switched between the solid mode, as a virtual wall, or liquid mode, which changes the perceivable viscosity when going below the surface level.

To render solids, a god-object method implementing a simple spring-damper impedance was used. Spring stiffness of up to 1000 N/m could be achieved without leading to significant instability. Adding force sensors at the attachment points (see Section 8.1) should further improve this.

**FIGURE 13:**
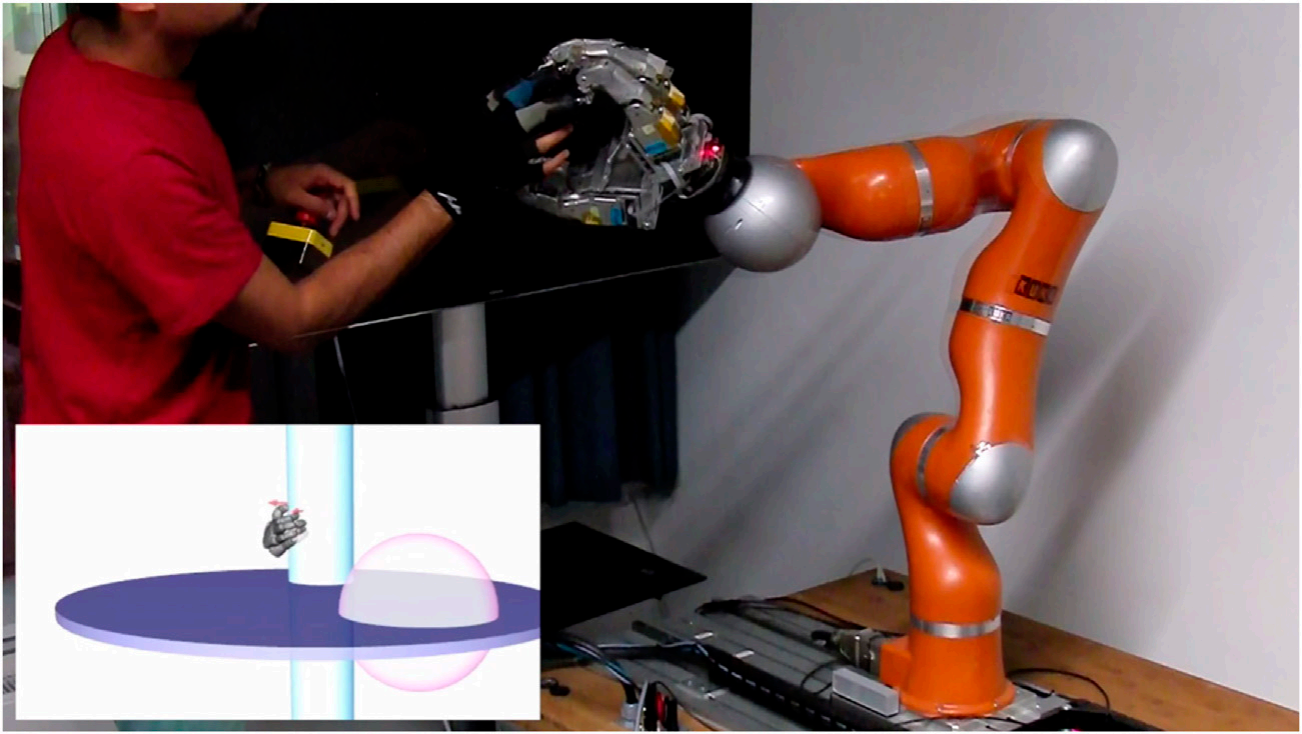
User interaction in a virtual environment with liquid and solid objects. The operator of the Exodex can be seen using a virtual hand in the virtual environment on screen (shown bottom left). The surface of a virtual fluid is represented by a purple disk. Solid bodies are rendered in the shape of a cylinder (in cyan) and a sphere (in pink). For a better view of the user interaction in the virtual reality environment, please refer to the Supplementary Video.

For the liquid media, only viscosity forces were rendered, which mainly stimulate proprioceptive and kinesthetic senses, while other fluid cues require cutaneous and temperature sensing. The viscosity forces were calculated based on a simplified drag equation (Schmidt et al., 2020). With this trade-off in accuracy, high enough frequencies could be achieved for the viscosity rendering to run simultaneously with the solid interaction loop. Despite the physical hardware limitations restricting the rendering of very low viscosity such as that of water, users were able to perceive media as fluid in the virtual environment and could clearly distinguish these from solids. In a user study (Schmidt et al., 2020), the viscosities between 1 and 30 Pa s were achieved stably on the Exodex. Especially in the higher viscosity range, the participants were able to distinguish different rendered fluid viscosities as in real-life experiments, with a Weber fraction of .3. Using Exodex as an UI, it was observed that virtual viscosity is mainly perceived through larger arm motions, e.g., as people do when checking the water in a bathtub, whereas more dexterous interactions with solid objects occur at the in-hand level. This suggests that the viscosity rendering could be mainly implemented in the robotic arm, while the robotic fingers are free to render crisp solid body interactions. Thus, exploration of more complex virtual environments is enabled, where both solids and fluids can be simultaneously rendered and explored as can be seen in Figure 13 and in the Supplementary Video.

### 7.2 Gesture-Commanded Telemanipulation

Earlier work has shown the gesture-driven approach to telecommand robotic end effectors using a data glove to be particularly effective for high dexterity tasks such as in-hand manipulation, while reducing the teleoperator’s workload (Lii et al., 2012). For the Exodex, a neural network–based gesture command concept has been implemented.

A two-layer neural network was trained on six hand gestures, with

P_1_: outstretched fingers,
P_2_: index finger pointing,
P_3_: diver’s “OK,”
P_4_: power-grasp, small object (e.g., size of an apricot),
P_5_: power-grasp, larger object (e.g., size of a grapefruit), and
P_6_: (attempt to) touch bottom of the little finger with thumb tip.

**FIGURE 14:**
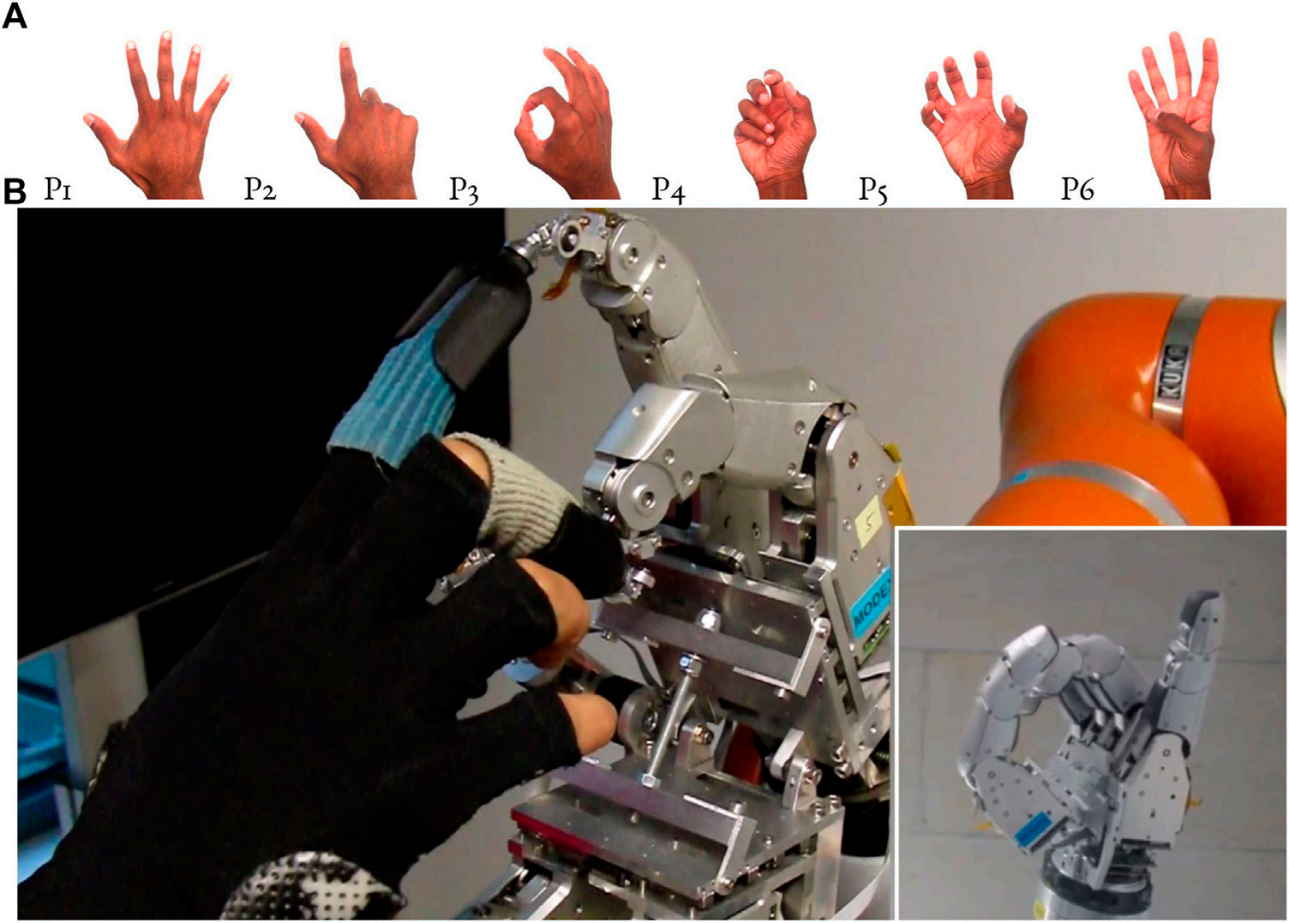
Gesture-driven hand teleoperation. **(A)** The different user hand gestures trained for recognition: left to right: outstretched fingers (P1), index finger pointing (P2), diver’s “OK” (P3), power-grasp, small object (P4), power-grasp, larger object (P5), touch bottom of little finger with thumb tip (P6). **(B)** An example of telecommanded gesture being executed by a DLR Five-Finger Hand (bottom right). More examples can be seen in the Supplementary Video.

The gesture recognition was tested as an input method to a graphical UI which controls the robot, as well as for telemanipulating another robot, as shown in Figure 14B. The gesture command in operation can be seen in the Supplementary Video.

To examine the ability to accommodate different hand sizes, we tested the gesture recognition on three adult females and two adult males, from a 15th percentile adult female hand span of 168 mm to an 86th percentile adult male at 205 mm (Greiner, 1991, p. 157). The device kinematics was optimized for each hand size as in Section 5. The gestures from Section 7.2 were used as the set C of required hand configurations, and we used gesture P5 as $C_{m}$ the (set of) gestures around which to maximize manipulability. In addition, we tried two children’s hand sizes with spans of 138 and 158 mm. Kinematics were optimized manually for the experiments with the child subjects, due to time constraints on running the optimization and the availability of the children.

Subjects had photographs in front of them showing the six positions in Figure 14A with their corresponding number. The trial progressed as follows:

(1) The subject practiced the six positions to get adjusted to the haptic UI.
(2) The number of each position was shown on screen for 16 s, during which time the subject had to form the corresponding position with their hand. All six positions were cycled through in order, twice.
(3) The second step was repeated, but the subject had visual feedback on screen of both the number of the recognized gesture, and a simulation of a teleoperated (robot) hand making the gesture.

Results are presented in Table 2. For each subject, we show which gestures were not achieved (on either attempt) either with or without visual feedback. We show whether this was within 8 s (the reaction time of subjects was sometimes several seconds, so a gesture being recognized within 8 s corresponds to a problem-free formation and recognition of the gesture) or within 16 s. The recognized gesture was counted only if it was constant for 2 s. Gesture P_6_ is difficult to form when connected to the exoskeleton. This is discussed in the following section. Gesture P_5_ is difficult for the neural network to tell apart from P_4_, as they are both power grasps.

**TABLE 2:** Gestures not recognized within 8 and 16 s, with and without visual feedback.

Subject	Gender^a^	Hand size	Gestures not achieved, no feedback^b^		Gestures not achieved, with feedback^b^	
		(mm)	( < 8 s)	( < 16 s)	( < 8 s)	( < 16 s)
Adult 1	F	168				
Adult 2	F	174	P5, P6	P5, P6	P6	
Adult 3	F	182	P6	P6	P6	
Adult 4	M	186				
Adult 5	M	205				
Child 1^c^	M	138	P5, P6	P5	P3, P6	
Child 2^c^	F	158				

a F, female; M, male.

b Only the gestures not achieved are listed in the interest of legibility.

c Exodex Adam configuration was manually optimized.

Interestingly, the child of hand size 158 mm was unable to achieve gesture P_6_ at all after the first attempt at manually optimizing the Exodex kinematics. When the kinematics were readjusted, the child could achieve all gestures. However, this suggests that 1) rather than a one-size-fits-all design, passive DOF allow the design to be optimized and function well for a range of hand sizes, and 2) manual optimization does not always yield a valid solution, further motivating the automated optimization in Section 5.

### 7.3 Teleoperation of a Dexterous Hand–Arm System

**FIGURE 15:**
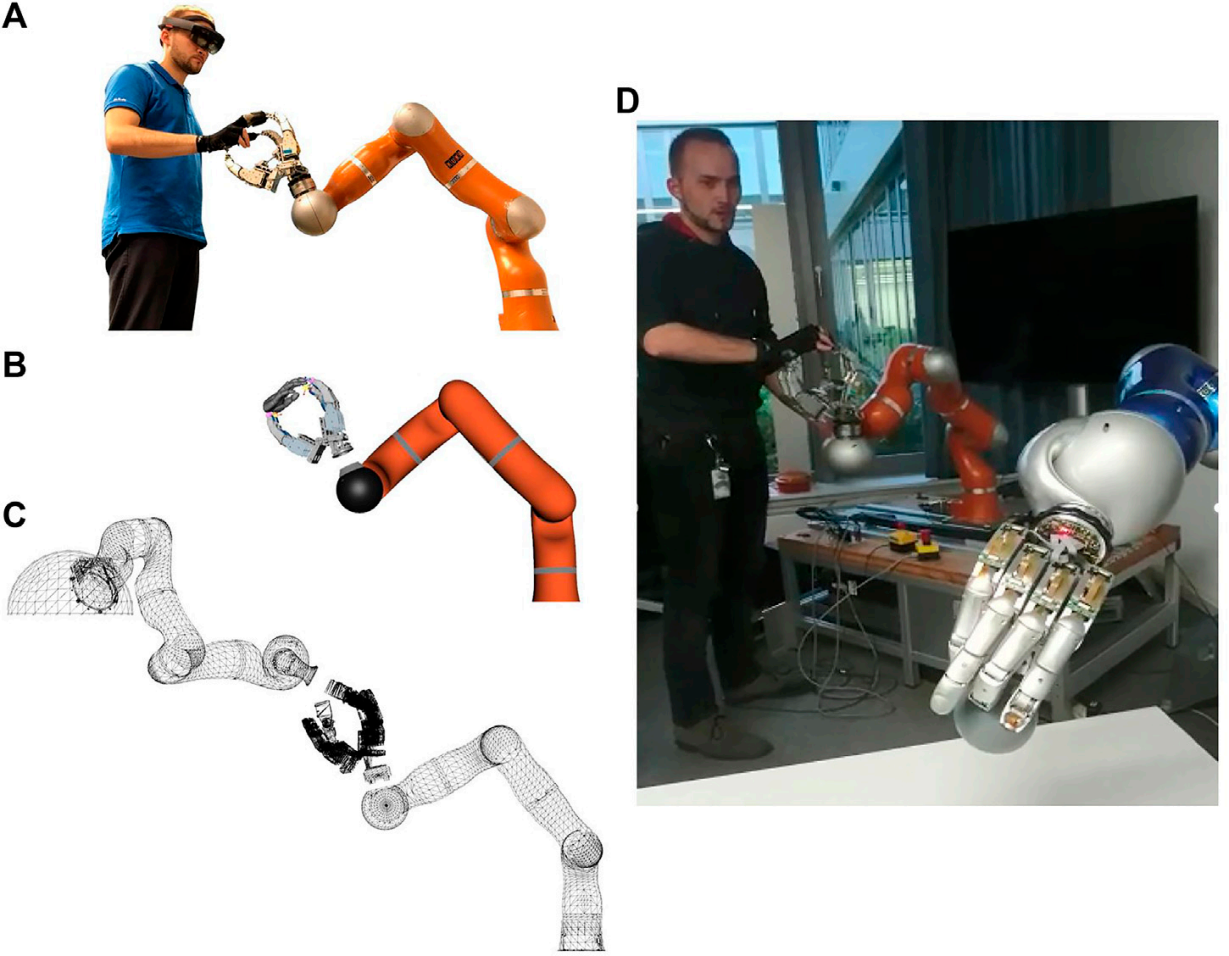
Exodex employed as a haptic UI to teleoperate a hand–arm system. **(A)** The operator’s hand is attached to Exodex. **(B)** The contact points of the hand and interface are calculated using the forward kinematics. The contact points are then used to estimate the human hand posture using the inverse kinematics in Section 6. **(C)** A joint-to-joint approximation of avatar robot hand is calculated to match the operator’s hand. **(D)** Exodex being used to teleoperate a hand–arm system to pick up an object.

The Exodex can also be used as an input device to teleoperate a real or virtual avatar robot (see Figure 15) with haptic and/or visual feedback. We used the Exodex to command a anthropomorphic hand–arm setup (see Figure 15) composed of a custom configured KUKA DLR-LWR 4+ (with joints reconfigured to match human arm kinematics) and a DLR Five-Finger hand with a two-channel teleoperation setup (Frisoli et al., 2004). The visual feedback from the remote environment plus additional task-specific information are provided through a Microsoft Hololens.

Both direct teleoperation and an MMT (Passenberg et al., 2010; Xu et al., 2016) approach were tested. In direct teleoperation, an impedance controller on the avatar tracks the input device in the task space. The haptic feedback displayed on the Exodex to the teleoperator’s hand is calculated based on environmental forces measured on the avatar. In our MMT approach, both the different kinematics of Exodex and avatar as well as possible communication delays are accounted for by a virtual model at the local site. The user interacts haptically directly with this intermediate model. Differences between the virtual model and the real avatar’s environment are reconciled using Dynamic Motion Primitives and Reinforcement Learning. More detailed description and evaluation are found in Beik-Mohammadi et al. (2020).

### 7.4 Summary and Discussion

Looking at the overall success of the different use cases deployed, we believe that the Exodex has fulfilled the functions of a haptic interface that we set out for. In a mixed media virtual environment, the users can interact with and distinguish different stiffness (e.g., like a drum-skin, dough, or air-balloon), shapes (i.e., plane, sphere, cylinder), and curvature. They were also able to differentiate between different media, such as solids and fluids of different viscosities. The users could also grasp and manipulate objects in a virtual world using intuitive exploration procedures. This set of capabilities shows great potential for applications such as underwater exploration.

Looking at a more abstract level of teleoperation through gestures, the Exodex was able to facilitate the recognition of the set of different gestures in the experiment, and in turn command the avatar robotic hand. This also helped validate our hand state and pose estimation approach presented in Sections 5, 6.

The successful teleoperation of a hand–arm robotic avatar through adaptive MMT demonstrates the Exodex’s ability to command a high complexity robot in performing different hand-–arm motion skills such as object handling. Furthermore, it shows the feasibility to delegate some autonomy (more of the lower level robotic tasks) to the avatar.

However, our first attempt at a whole-hand haptic UI still has shortcomings that can be addressed. Firstly, there was no tactile perception, as the fingers were inside silicone sleeves. This gives the user a somewhat insulated feel, even with force reflection. The experience can be likened to exploring the physical world with gloves on. Furthermore, although the finger sleeves are usually comfortable to wear, with a limited number of sizes, the fit is not always perfect. This can sometimes cause unwanted pressure on the distal links of the user’s finger. If the rigid thimble part of the finger sleeve was too tight, this could be uncomfortable over longer periods of time. An adjustable finger sleeve, or more gradations in the size can address the discomfort.

Secondly, the operator of the Exodex should be able to move their hand and arm in space freely, feeling as little of the mechanism’s inertia and friction as possible. To reduce the inertia, feed-forward control was implemented (see Section 6.5) and to reduce the friction in the fingers, a friction observer was implemented (see Section 6.4). Friction in the LWR is barely perceptible, and so was not compensated. Despite these measures, inertia and friction effects can still be felt. Since excessively high feed-forward gains lead to instability in the fingers, it is not possible to completely remove the feeling of inertia by increasing feed-forward gain. Sometimes, users had to use their other hand to move the LWR or readjust the Exodex. Reducing the weight (the current version weighs approximately 2.5 kg) and moment of inertia of the Exodex can help. Additional force/torque sensors at the attachment points can also help improve the control performance to reduce unwanted perceivable dynamics.

Thirdly, the workspace of the robotic fingers of the Exodex is limited, and this led to a compromise, as detailed in Section 4, of ensuring that the most useful human hand positions for grasping and manipulation were within the workspace. However, other hand positions were difficult, e.g., P_6_ in Section 7.2, or impossible. Since the workspace of the human’s thumb is so large, some thumb positions (e.g., full flexion), lie outside the workspace of the robotic finger. We observed that subjects would sometimes contort their hands to achieve the gesture despite the constraints of the system’s mechanics.

The reason gesture P_6_ (see Section 7.2) was problematic was its difficulty to be detected, even when achievable. In this position, some of the human hand joints were near their joint limits. Since the null space optimization of the human hand kinematics estimation (see Section 6.3) optimizes away from joint angles, it could be that this makes P_6_ more difficult for the algorithm to reconstruct the angles of the human hand joints accurately.

Another drawback, related to the limited workspace, was that joint limits of the LWR and of the Exodex’s robotic fingers are often reached. To protect the joints from damage, we implemented a virtual impedance (spring-damper system) at the joint limits which prevents the user from reaching the physical limits. However, this may be perceived as a virtual object by the user, or may simply be confusing.

To address this, a variety of solutions are possible. On the control side, virtual forces pulling away from joint limits could be projected into the null space with a suitable dynamic null space projector (Dietrich et al., 2015), reorienting the device away from the joint limits without exerting forces at the attachment points. Another solution is to limit the workspace in which the human operates, and instead use metaphor 3D interaction techniques such as go-go (Poupyrev et al., 1996) or ray-casting (Mine et al., 1997) as proposed in Bowman and Hodges (1997) and Ouramdane et al. (2006), to allow the user to reach objects beyond their physical workspace. Concretely, the users are lead to believe—via visual cues—that their hand has reached a position, which is in reality unattainable while attached to the haptic UI.

In the Exodex, the forces exerted at the center of the gimbal are the same as those exerted on the human. However, there is often a small torque induced since the distance from the attachment point to the human is often not parallel to the force acting at the gimbal. In our design, we kept these torques to a minimum by keeping the attachment point as close to the gimbal as was mechanically possible. Nevertheless, this inherent issue of the human–Exodex attachment should be examined with new attachment concepts. This, along with other drawbacks that we discovered during this work can be addressed in our continuing development on the Exodex.

One key feature omitted in this first version of Exodex is the dedicated haptic interaction for the ring and little fingers, which we plan to add in the follow-up version. Nevertheless, users were able to perform the necessary precision and power grasps without feedback to the ring and little fingers. They would generally hold these fingers in full flexion when power-grasping and extend them when precision grasping. Feix et al. (2016) have shown that some power grasps can be performed with the thumb, index, and middle fingers and the palm. However, adding two more robotic fingers to Exodex would finally enable a full(er) variety of grasps and in-hand manipulations.

By comparison, a fingertip-only system, such as the HIRO, gives the user more possibilities to move the UI through its workspace. A somewhat apt analogy is our operation of a steering wheel, where the user can take the hand off the wheel, then place it back on to regain range of motion on the user side, and continue manipulating the input device. With Exodex, this whole-hand attachment scheme takes away this possibility. What it gains, in turn, is the whole-hand immersive interaction. One can say that although the HIRO is more of an input style suitable to manipulating the input device, the Exodex’s strength lies in its close coupling of the whole user’s hand. As a first version of integrated system from our ground whole-hand haptic UI system, the Exodex succeeded in providing a safe and comfortable operation, immersive user experience, user hand pose capturing, and enabling several command modalities. This gave us the confidence to continue forward with it as a capable device for a wide array of applications.

## 8 Conclusion

We present a novel whole-hand haptic input device, the Exodex Adam, a front-facing, mirror attachment haptic UI for the hand. Attached to the user at the fingers and the palm, it not only allows tracking of the human hand pose but also offers an immersive haptic experience. This enables the rendering of forces from power grasps, precision grasp, in-hand manipulation, as well as whole-hand exploration of a remote or virtual environment.

Through the deployment of the Exodex in different use cases, we validated our haptic UI concept’s viability for a wide array of applications. Its combination of force reflection and whole-hand interaction enables the user to intricately interact with complex environments. We also showcased its effectiveness in different command modalities, making it a viable system for shared control strategies and scalable autonomy based telerobotics. The reconfigurable design has been shown to safely accommodate users of different hand sizes and shapes.

We believe with the current Exodex, and its further development, such a haptic UI system shall serve a growing array of fields and applications from space and underwater exploration, physical therapy, or rehabilitation, VR activities, to an assortment of telerobotic applications.

### 8.1 Future Work

As already discussed in Section 7.4, the most immediate improvement to the Exodex is the addition of dedicated haptic interaction for the ring and little fingers, by adding two more robotic fingers to the system. Furthermore, by automating the palm base’s adjustable DOF with actuation and sensorization, the robotic fingers’ base pose and position can be automatically generated, thus streamlining the hand pose estimation. It may also be possible to further incorporate the actuated palm base DOF into the motion of the Exodex to increase the active workspace during operation.

Currently, the Exodex only stimulates proprioceptive and kinesthetic perception (i.e., by rendering forces), which are only two components of haptic feedback. In future development, miniaturized vibrotactile actuators in the finger sleeves may be able to provide additional cutaneous feedback (Bolanowski Jr et al., 1988; Biswas, 2015). Adding tactile or cutaneous feedback (i.e., feeling warmth, texture, etc. on the skin) as part of future work would further enrich user experience.

Better estimation of the human hand pose would allow us to apply more accurate rendering algorithms taking into account the different frictional and mechanical properties of the human hand. An area of future work that should be continued is the implementing and testing of these rendering algorithms.

Although Exodex’s current implementation already allows good hand pose estimation, its accuracy can be improved by adding angular sensors on each of the DOF on the gimbal. Furthermore, by introducing force sensors at the attachment points, we could acquire more detailed measurements of the applied force from the user, which can in turn improve feed-forward control performance.

Finally, this work demonstrates the broad usability of the Exodex with different command modalities. Another urgent next step is to identify more specifically relevant applications in need of such a capable hand-arm haptic UI, so that development can focus on their specific requirements.

## Data Availability

The raw data supporting the conclusion of this article will be made available by the authors, without undue reservation.
